# Predicting seizure outcome after epilepsy surgery: Do we need more complex models, larger samples, or better data?

**DOI:** 10.1111/epi.17637

**Published:** 2023-06-16

**Authors:** Maria H. Eriksson, Mathilde Ripart, Rory J. Piper, Friederike Moeller, Krishna B. Das, Christin Eltze, Gerald Cooray, John Booth, Kirstie J. Whitaker, Aswin Chari, Patricia Martin Sanfilippo, Ana Perez Caballero, Lara Menzies, Amy McTague, Martin M. Tisdall, J. Helen Cross, Torsten Baldeweg, Sophie Adler, Konrad Wagstyl

**Affiliations:** ^1^ Developmental Neurosciences Research & Teaching Department UCL Great Ormond Street Institute of Child Health London UK; ^2^ Department of Neuropsychology Great Ormond Street Hospital London UK; ^3^ Department of Neurology Great Ormond Street Hospital London UK; ^4^ The Alan Turing Institute London UK; ^5^ Department of Neurosurgery Great Ormond Street Hospital London UK; ^6^ Department of Neurophysiology Great Ormond Street Hospital London UK; ^7^ Clinical Neuroscience Karolinska Institute Solna Sweden; ^8^ Digital Research Environment Great Ormond Street Hospital London UK; ^9^ North Thames Genomic Laboratory Hub Great Ormond Street Hospital London UK; ^10^ Department of Clinical Genetics Great Ormond Street Hospital London UK; ^11^ Young Epilepsy Lingfield UK; ^12^ Imaging Neuroscience UCL Queen Square Institute of Neurology London UK

**Keywords:** epilepsy surgery, machine learning, pediatric, prediction

## Abstract

**Objective:**

The accurate prediction of seizure freedom after epilepsy surgery remains challenging. We investigated if (1) training more complex models, (2) recruiting larger sample sizes, or (3) using data‐driven selection of clinical predictors would improve our ability to predict postoperative seizure outcome using clinical features. We also conducted the first substantial external validation of a machine learning model trained to predict postoperative seizure outcome.

**Methods:**

We performed a retrospective cohort study of 797 children who had undergone resective or disconnective epilepsy surgery at a tertiary center. We extracted patient information from medical records and trained three models—a logistic regression, a multilayer perceptron, and an XGBoost model—to predict 1‐year postoperative seizure outcome on our data set. We evaluated the performance of a recently published XGBoost model on the same patients. We further investigated the impact of sample size on model performance, using learning curve analysis to estimate performance at samples up to *N* = 2000. Finally, we examined the impact of predictor selection on model performance.

**Results:**

Our logistic regression achieved an accuracy of 72% (95% confidence interval [CI] = 68%–75%, area under the curve [AUC] = .72), whereas our multilayer perceptron and XGBoost both achieved accuracies of 71% (95% CI_MLP_ = 67%–74%, AUC_MLP_ = .70; 95% CI_XGBoost own_ = 68%–75%, AUC_XGBoost own_ = .70). There was no significant difference in performance between our three models (all *p* > .4) and they all performed better than the external XGBoost, which achieved an accuracy of 63% (95% CI = 59%–67%, AUC = .62; *p*_LR_ = .005, *p*_MLP_ = .01, *p*_XGBoost own_ = .01) on our data. All models showed improved performance with increasing sample size, but limited improvements beyond our current sample. The best model performance was achieved with data‐driven feature selection.

**Significance:**

We show that neither the deployment of complex machine learning models nor the assembly of thousands of patients alone is likely to generate significant improvements in our ability to predict postoperative seizure freedom. We instead propose that improved feature selection alongside collaboration, data standardization, and model sharing is required to advance the field.

**Key Points**

- We trained three models – a logistic regression, a multilayer perceptron, and an XGBoost model – to predict seizure outcome and found that they performed equally well (AUC = .70‐.72).
- We applied a previously published machine learning model to our center’s patients and found that it underperformed (AUC = .62 on our cohort vs AUC = .73‐.74 on the original cohorts).
- Expanding our cohort beyond its current size, up to sample sizes of N = 2000, would not provide substantial gains in model performance.
- We were able to improve model performance through data‐driven feature selection.
- Future improvements in our ability to predict outcome will require improved feature selection, collaboration between epilepsy surgery services, data standardization, and model sharing.

## 1 INTRODUCTION

Despite careful evaluation, up to one third of patients with drug‐resistant epilepsy are not rendered seizure‐free through surgery.^1^ This underscores the need to identify which patients are likely to benefit from surgery before the intervention has been carried out.

Surgical candidate selection is typically decided by a multidisciplinary team. This form of expert clinical judgment relies on experience and available evidence, and achieves a moderate degree of accuracy when predicting surgical success.^2^ To aid clinical judgment, some studies have reported average estimates of seizure freedom for specific types of epilepsy (e.g., temporal lobe epilepsy).^1^ Other studies have focused on identifying multiple predictors of postoperative seizure outcome, without taking into account how these predictors may interact.^1^

In an effort to synthesize patient characteristics and provide objective predictions of seizure freedom, researchers have developed statistical models and calculated risk scores that can generate individualized predictions of outcome.^3^, ^4^, ^5^ These have included the Epilepsy Surgery Nomogram,^3^ the modified Seizure Freedom Score,^4^ and the Epilepsy Surgery Grading Scale.^5^ These tools do not, however, perform better than clinical judgment.^2^, ^6^ Researchers are, therefore, increasingly turning to machine learning in an attempt to improve prediction accuracy.

Machine learning is being leveraged within the realm of clinical research at an exponential pace. The epilepsy surgery pathway generates a plethora of diverse data. As such, it would seem to create an ideal opportunity for the application of machine learning technology. Several machine learning models have indeed been developed to date to predict seizure outcome.^7^, ^8^, ^9^, ^10^, ^11^, ^12^, ^13^, ^14^, ^15^, ^16^, ^17^, ^18^, ^19^, ^20^, ^21^, ^22^, ^23^, ^24^, ^25^, ^26^, ^27^, ^28^, ^29^ The majority of these models have, however, been trained on relatively small sample sizes (*N* < 100)^7^, ^8^, ^10^, ^11^, ^13^, ^14^, ^15^, ^16^, ^18^, ^19^, ^20^, ^22^, ^24^, ^25^, ^26^, ^27^, ^28^, ^29^ and, therefore, have a high risk of “overfitting” (a model overfits when it models the training data set too closely, performing well on this data set but consequently underperforming on new, “unseen” data sets).^30^, ^31^ Model training sets have also been composed almost exclusively of temporal lobe surgery patients,^8^, ^9^, ^10^, ^11^, ^13^, ^14^, ^15^, ^16^, ^17^, ^18^, ^21^, ^22^, ^23^, ^24^, ^25^, ^26^, ^27^, ^29^ often relied on postoperative factors,^7^, ^10^, ^12^, ^23^, ^25^, ^26^, ^27^ and frequently utilized postprocessing neuroimaging analyses that cannot be replicated readily by others.^11^, ^13^, ^14^, ^15^, ^16^, ^17^, ^18^, ^20^, ^21^, ^25^, ^27^, ^28^, ^29^ As such, many existing models may be difficult to incorporate into routine preoperative evaluation. Perhaps more importantly still, none of these models have been externally validated on a substantial cohort.^17^, ^20^ It is, therefore, unknown how well they would perform if used by another surgery center, and whether their adoption as a replacement for traditional statistical modeling approaches is justified.

**FIGURE 1:**
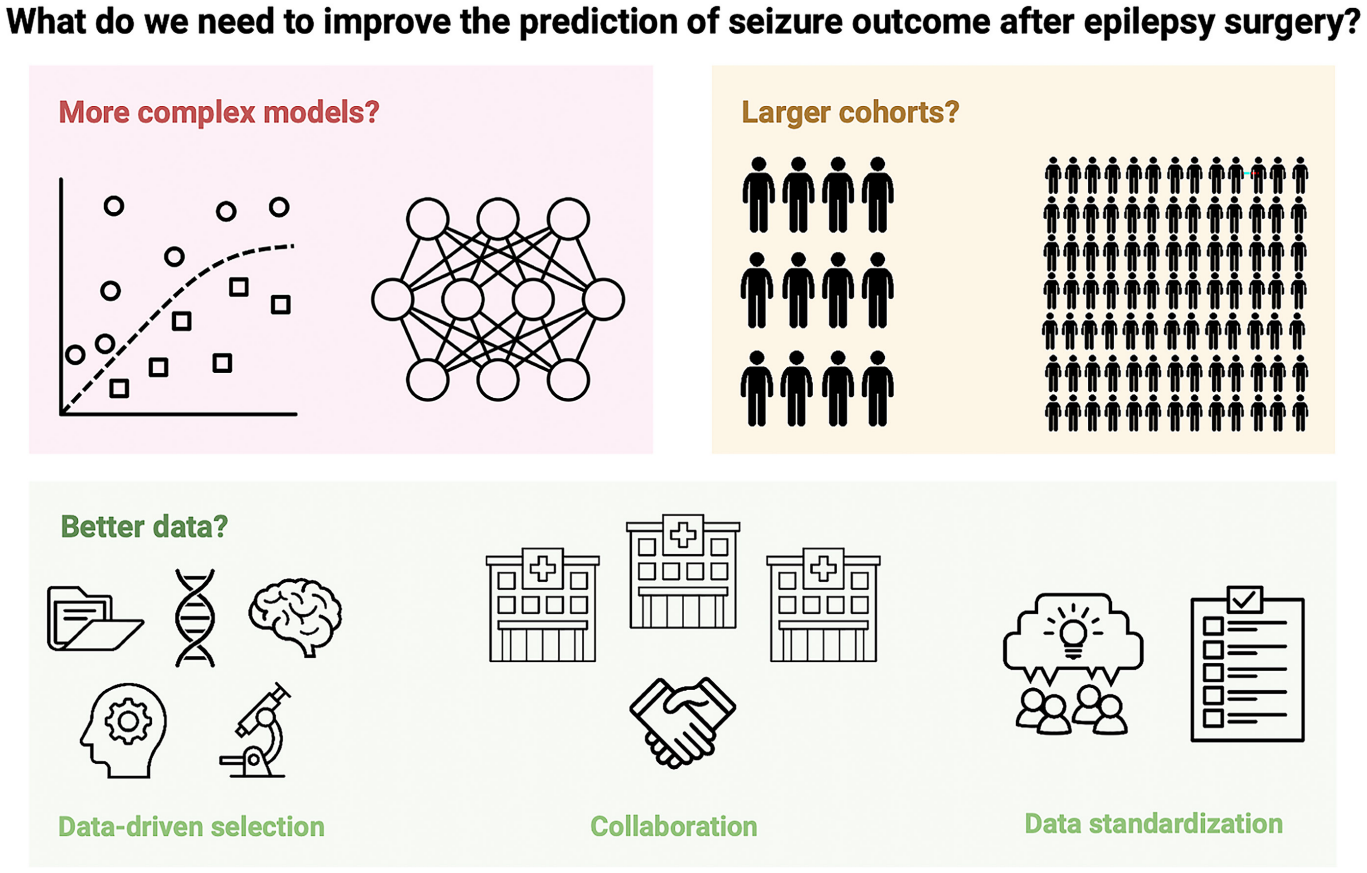
Study overview. We investigated the impact of model type, sample size, and feature selection on our ability to accurately predict postoperative seizure outcome.

To advance this field, we asked whether (1) more complex models, (2) larger sample sizes, or (3) better selection of clinical predictors would improve our ability to predict postoperative seizure outcome (Figure 1). To address the first question, we trained three different models—a traditional logistic regression and two machine learning models—to predict seizure outcome on our data set. We also tested the performance of an external, pre‐trained machine learning model^12^ on our data set and compared its performance to that of our models. To address the influence of sample size, we investigated how varying sample size—both within and extrapolating beyond our current cohort—impacted model performance. To address the influence of number and type of clinical predictors, we investigated how the inclusion of different predictors affected model performance.

## 2 MATERIALS AND METHODS

### 2.1 Patient cohort

We retrospectively reviewed medical records for all children who underwent epilepsy surgery at Great Ormond Street Hospital (GOSH; London, UK) from January 1, 2000, through December 31, 2018. We included patients who underwent surgical resection or disconnection. We excluded palliative procedures (corpus callosotomy and multiple subpial transections), as well as neuromodulation (deep brain stimulation and vagus nerve stimulation) and thermocoagulation procedures. If patients had undergone multiple surgeries over the course of the study period, we included only their first surgery.

### 2.2 Data set description

We retrieved medical records and extracted the following information: patient demographics, epilepsy characteristics, preoperative magnetic resonance imaging (MRI) findings, preoperative interictal and ictal electroencephalography (EEG) characteristics, preoperative antiseizure medication (ASM; including both total number of ASM trialed from time of epilepsy onset to time of preoperative evaluation, as well as number of ASM at time of preoperative evaluation), surgery details, genetic results, and histopathology diagnosis. A complete list of variables extracted and information about how we categorized these data can be found in Appendix S1.

We classified patients as either seizure‐free (including no auras) or not seizure‐free at 1‐year postoperative follow‐up. We also recorded if patients were receiving, weaning, or off ASMs at this time point.

### 2.3 Statistical analysis

We calculated the descriptive statistics for the cohort and presented these using mean with standard deviation, median with interquartile range, and count with proportion, as appropriate.

We checked if continuous data were normally distributed using Shapiro–Wilk tests.^32^ None of the continuous variables were normally distributed. We, therefore, investigated associations between demographic, clinical, and surgical variables using the Mann–Whitney *U*, Kruskal–Wallis *H*, chi‐square test of independence, and Spearman's rank correlation coefficient, as appropriate. All tests were two‐tailed, and we set the threshold for significance a priori at *p* < .05. We corrected for multiple comparisons using the Holm method.^33^

We performed univariable logistic regression analyses to investigate which clinical variables predicted seizure outcome at 1‐year postoperative follow‐up. In the case of categorical variables, the group known to have the highest seizure freedom rate (according to past literature) was used as the reference category. All other groups were then compared to this reference category to determine if they were significantly less (or more) likely to achieve seizure freedom through surgery. For example, “unilateral MRI abnormalities” was selected as the reference category for the categorical variable “MRI bilaterality,” and we investigated whether those with “bilateral MRI abnormalities” were significantly less (or more) likely to be seizure‐free after surgery. We again corrected for multiple comparisons using the Holm method.^33^

#### 2.3.1 Effect of model type on model performance

We performed a multivariable logistic regression (LR) with independent variables that (1) could be obtained preoperatively and (2) were found to be predictive of seizure outcome. We developed a second version of this model, in which MRI diagnosis was replaced with histopathology diagnosis, to determine if this affected model performance.

We used the same predictors to train two machine learning models: a multilayer perceptron (MLP) and an XGBoost model. We chose an MLP due to its high predictive performance, allowing for nonlinear interactions between input variables. We trained the MLP with two hidden layers, with 5 and 10 hidden neurons respectively, balancing the need for sufficient complexity to learn feature interactions across multiple features, while limiting the capacity of the network to overfit to the training data. We chose an XGBoost model to ensure that we could compare the performance of this to the performance of the XGBoost model published by Yossofzai et al.^12^

After training our own three models, we applied the XGBoost model by Yossofzai et al.^12^ to the same patient cohort. We evaluated the performance of all models using stratified 10‐fold cross‐validation. We used a stratified approach to address the outcome imbalance observed in our cohort. We calculated the null accuracy (the accuracy the model would achieve if it always predicted the more commonly occurring outcome in our cohort, i.e., seizure‐free), the tested model accuracy, and the area under the receiver‐operating characteristic (ROC) curve (AUC) for each model. We reported both the mean AUC obtained across all 10 folds as well as the AUC obtained from each individual fold. We compared the accuracies of the respective models using McNemar's test.

#### 2.3.2 Effect of sample size on model performance

We investigated how sample size affected model performance by using a previously described learning curve analysis approach.^34^ First, we trained our models on 38 different sample sizes, starting at *N* = 20 and finishing at *N* = 700 patients. At each sample size, we evaluated model performance, specifically model accuracy. This allowed us to create a learning curve, plotting model performance against sample size. We then chose an inverse power law function to model the learning curve. We used this function to predict model performance on expanded sample sizes of up to *N* = 2000.

#### 2.3.3 Effect of clinical predictors on model performance

We explored how the number of included predictors, as well as their nature, affected model performance. We used the coefficients from our univariable logistic regression analyses to determine how informative different predictors were. We then added significant predictors one‐by‐one into our models, from the most informative to the least informative. At each point, we plotted model AUC and confidence intervals (CIs; obtained across the 10 folds).

We performed all statistical analyses and visualizations in Python version 3.7.2 and R version 3.6.3. Our MLP and XGBoost models were implemented using the scikit‐learn library.^35^ The study’s analytic code is available on GitHub (https://github.com/MariaEriksson/Predicting‐seizure‐outcome‐paper).

## 3 RESULTS

### 3.1 Patient cohort

A total of 797 children were identified as having undergone first‐time surgical resection or disconnection. Demographic information and clinical characteristics for these patients are displayed in Table S1. Data relating to semiology (past seizures and seizures at time of preoperative evaluation) as well as interictal and ictal EEG characteristics are displayed in Table S2. Genetic diagnoses are listed in Tables S3 and S4.

Seizure outcome at 1‐year follow‐up was available for 709 patients, of which 67% were seizure‐free. Of these, 51% were receiving ASM, 34% were weaning ASM, and 15% were not receiving ASM.

### 3.2 Relationships between variables

**FIGURE 2:**
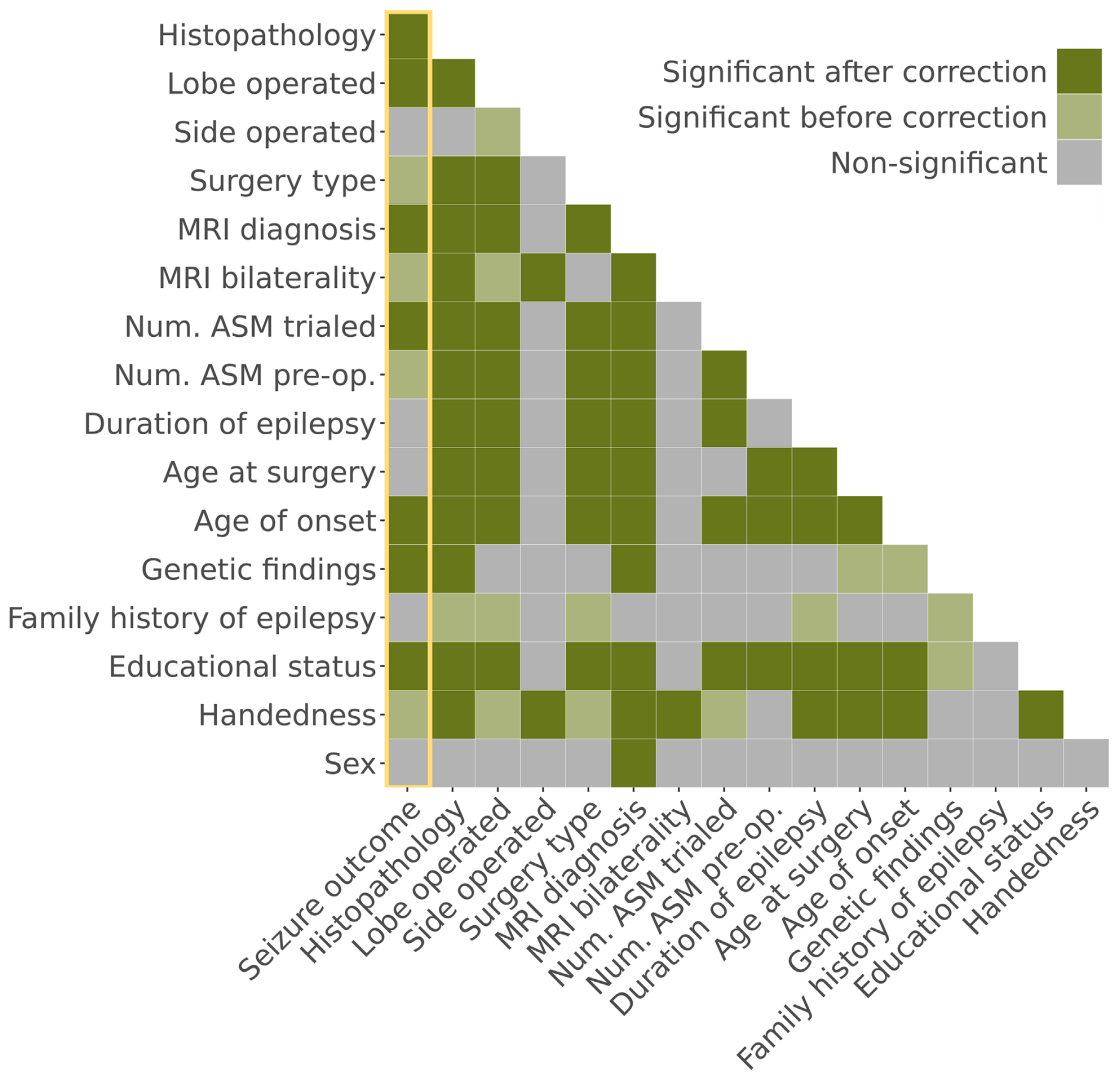
Relationships between demographic, clinical, and surgical variables. Relationships are shown both before and after correction for multiple comparison using the Holm method. We have highlighted relationships with seizure outcome using a yellow box. ASM, antiseizure medication; Num. ASM pre‐op, number of antiseizure medications at time of preoperative evaluation; Num. ASM trialed, total number of different antiseizure medications trialed from epilepsy onset to preoperative evaluation.

Relationships between demographic, clinical, and surgical variables are displayed in Figure 2. Full statistics are reported in Table S5.

### 3.3 Univariable logistic regression analyses

Univariable logistic regression analyses identified the following features as predictive of 1‐year postoperative seizure freedom: handedness, educational status, genetic findings, age of epilepsy onset, history of infantile spasms, spasms at time of preoperative evaluation, number of seizure types at time of preoperative evaluation, total number of ASMs trialed (from time of epilepsy onset to time of preoperative evaluation), MRI bilaterality (unilateral vs bilateral MRI abnormalities), MRI diagnosis, type of surgery performed, lobe operated on, and histopathology diagnosis (Table S6).

### 3.4 Effect of model type on model performance

#### 3.4.1 Logistic regression models

Our multivariable LR achieved an accuracy of 72% (95% CI = 68%–75%) and an AUC of .72 (range across the 10 folds: .64–.82). When we assessed whether substituting MRI diagnosis with histopathology diagnosis would improve model performance, we found that this alternative LR achieved a similar accuracy of 73% (95% CI = 69%–79%; AUC = .72; range across the 10 folds: .60–.77). There was no significant difference in performance between the LR that included MRI diagnosis and the LR that included histopathology diagnosis (McNemar's test, chi‐square = .1, *p* = .8). This was likely due to the high degree of overlap between MRI and histopathology diagnoses (Figure S1).

#### 3.4.2 Multilayer perceptron and XGBoost models

Our MLP achieved an accuracy of 71% (95% CI = 67%–74%) and an AUC of .70 (range across the 10 folds: .63–.82). Our XGBoost also achieved an accuracy of 71% (95% CI = 68%–75%) and an AUC of .70 (range across the 10 folds: .62–.83).

#### 3.4.3 External XGBoost model

When we applied the XGBoost model developed by Yossofzai et al.^12^ to our data, it achieved an accuracy of 63% (95% CI = 59%–67%) and an AUC of .62.

#### 3.4.4 Comparison of model performances

**FIGURE 3:**
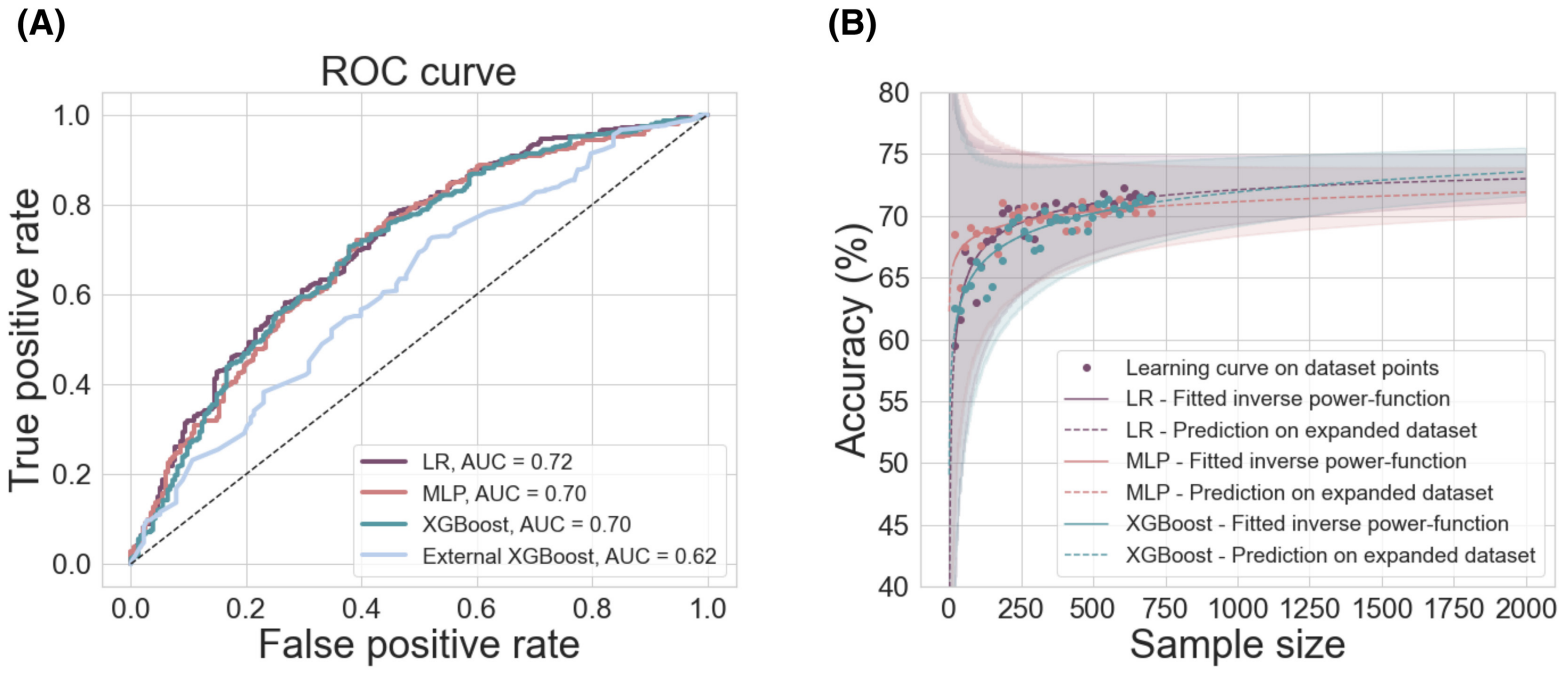
Impact of model type and sample size on model performance. (A) Receiver‐ operating characteristic (ROC) curves showing model performances. There was no significant difference in performance between our LR (purple), MLP (pink), and XGBoost (teal) models. All of our models performed significantly better than the XGBoost model recently developed by Yossofzai et al.^12^ (light blue). (B) The effect of sample size on model performance (accuracy). There was an improvement in model performance with increasing sample size for our LR, MPL, and XGBoost models, but only up until a certain point. After this, the models showed only marginal gains in performance. Extrapolating performance for sample sizes up to *N* = 2000 did not predict substantial improvement in model performance for any of our models. AUC, area under the (ROC) curve; LR, logistic regression; MLP, multilayer perceptron; ROC, receiver‐operating characteristic.

The AUCs of the respective models are compared in Figure 3A. There was no significant difference in performance between our LR and MLP (McNemar's test, chi‐square = .8, *p* = .4), our LR and XGBoost (McNemar's test, chi‐square = .1, *p* = .8), or our MLP and XGBoost (McNemar's test, chi‐square = .1, *p* = .8).

All three models performed better than the external XGBoost model (McNemar's test_LR_, chi‐square = 8.0, *p* = .005; McNemar's test_MLP_, chi‐square = 6.4, *p* = .01; McNemar's test_XGB own_, chi‐square = 6.8, *p* = .01). Our LR, MLP, and XGBoost models also performed significantly better than model null accuracy (McNemar's test_LR_, chi‐square = 8.7, *p* = .003; McNemar's test_MLP_, chi‐square = 5.3, *p* = .02; McNemar's test_XGB own_, chi‐square = 7.6, *p* = .006), whereas the external XGBoost model did not (McNemar's test_XGB external_, chi‐square = .6, *p* = .4).

### 3.5 Effect of sample size on model performance

Increasing our sample size within the limits of our cohort improved the performances of all our models (Figure 3B). However, visual inspection of model performance at increasing sample sizes showed that model performance started to plateau at around *N* = 400, after which point increases in sample size followed the law of diminishing returns. In the case of our LR, an increase from *N* = 20 to *N* = 120 led to a .08 increase in AUC (AUC = .593 vs AUC = .674). However, corresponding increases of 100 patients, from *N* = 200 to *N* = 300 patients and from *N* = 300 to *N* = 400 patients, led to .01 and <.01 increases in AUC, respectively (AUC = .689 vs .699 and AUC = .699 vs AUC = .705). Expanding our cohort beyond its current size, up to *N* = 2000, did not substantially improve the performances of any of our models (Figure 3B).

### 3.6 Effect of data inclusion on model performance

**FIGURE 4:**
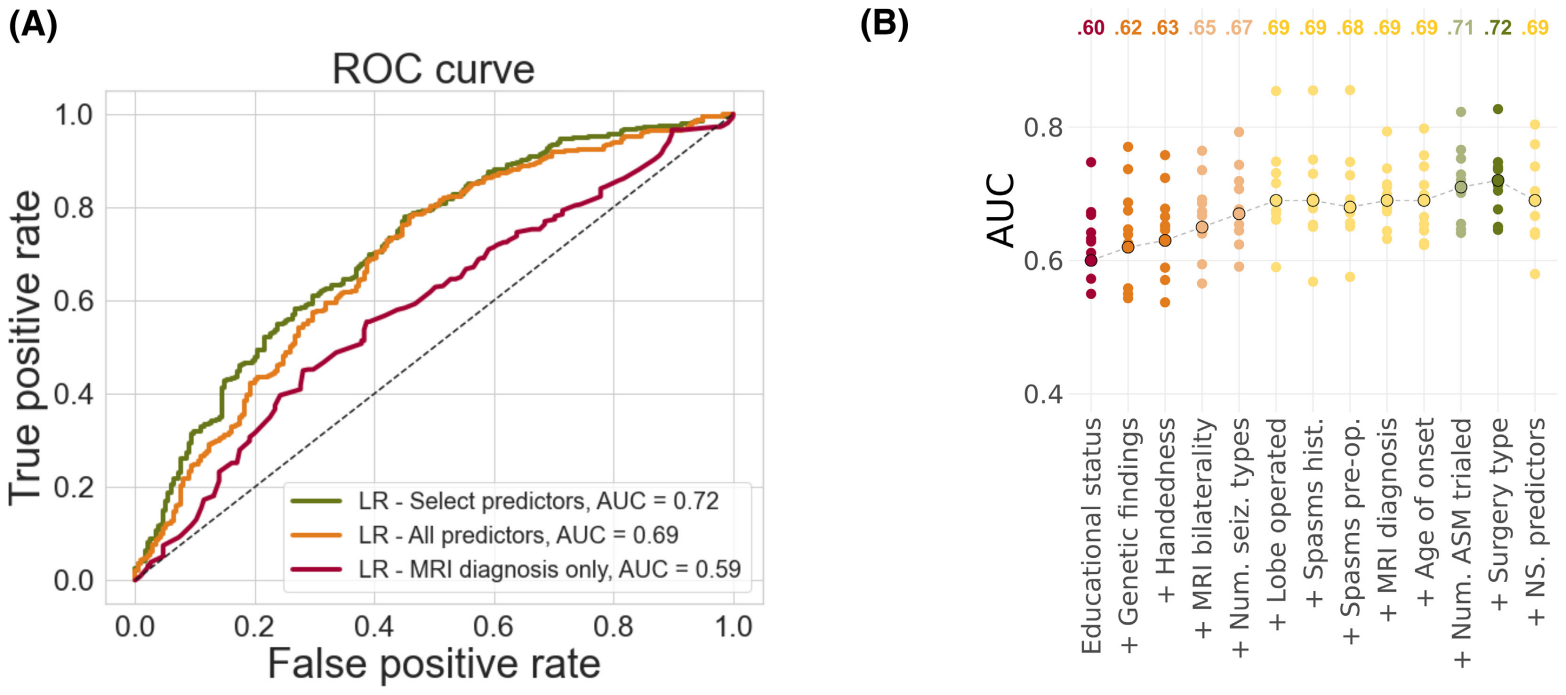
Impact of feature selection on model performance. (A) Receiver‐operating characteristic (ROC) curves showing model performance for our LR models containing (1) only MRI diagnosis (red), (2) all predictors (orange), and (3) predictors identified through data‐driven feature selection (green). Data‐driven selection involved including only predictors that were significantly predictive of 1‐year postoperative seizure outcome as identified in univariable logistic regression analyses. Corresponding ROC curves showing model performances for our MLP and XGBoost models are displayed in Figures S2 and S3. (B) Effect of data‐driven feature selection on model performance (AUC). Variables found to be significantly predictive of seizure outcome from univariable logistic regression analyses were added to the LR, from most information to least informative according to their coefficients. Model performance was best when all significantly predictive features were included in the model. Adding the remaining predictors collected for the study, that is, those that were not significantly predictive of seizure outcome, worsened model performance (far right). Points circled in black represent mean AUC obtained across all 10 folds. Noncircled points represent the AUCs obtained from each of the individual 10 folds. ASM, antiseizure medication; AUC, area under the (ROC) curve; LR, logistic regression; NS. predictors, non‐significant predictors; Num. ASM trialed, total number of different antiseizure medication trialed from epilepsy onset to preoperative evaluation; Num. seiz. types, number of seizure types at time of preoperative evaluation; ROC, receiver‐operating characteristic; Spasms hist., history of spasms; Spasms pre‐op, spasms at time of preoperative evaluation.

We found that adding more predictor features improved the performances of all models (Figure 4A and Figures S2 and S3). However, the greatest accuracy was achieved when data‐driven feature selection was used to filter which clinical predictors should be included in the models (i.e., when the models included only the variables that were found to be significantly predictive of seizure outcome in our univariable logistic regression analyses; Figure 4B). When we added variables that were not significantly predictive of seizure outcome in our univariable logistic regression analyses, model performance worsened (Figure 4B).

## 4 DISCUSSION

Up to one third of patients do not achieve seizure freedom through epilepsy surgery despite careful evaluation.^1^ There has been a longstanding history of trying to identify these patients preoperatively, both through traditional statistical modeling approaches and more complex machine learning techniques.^3^, ^4^, ^5^, ^7^, ^8^, ^9^, ^10^, ^11^, ^12^, ^13^, ^14^, ^15^, ^16^, ^17^, ^18^, ^19^, ^20^, ^21^, ^22^, ^23^, ^24^, ^25^, ^26^, ^27^, ^28^, ^29^ These attempts have, however, had limited success. In this study, we explored if we could improve our ability to predict seizure outcome by training more complex models, recruiting larger training sample sizes, or incorporating more or different types of clinical predictors.

To investigate the effect of model type on our ability to predict seizure outcome, we trained three different models, a logistic regression (or LR) and two machine learning models—a multilayer perceptron (or MLP) and an XGBoost—on the same cohort. We showed that our LR performed as well as our MLP and XGBoost models. We also applied a recently published XGBoost model by Yossofzai et al.^12^ to our cohort and found that this model performed worse than our models (AUC = .62 vs AUC = .70–.72). It also performed worse on our cohort compared to the cohorts it was trained and tested on (AUC = .62 vs AUC = .73–.74).

To address the value of larger patient sample sizes, we investigated model performance on a range of sample sizes, up to *N* = 2000. We found that the performances of all models improved until around *N* = 400, after which point they began to plateau.

To address the influence of clinical predictors, we varied both the number of predictors included in the models as well as the nature of these predictors. We demonstrated that using data‐driven feature selection (i.e., including only variables that were predictive of seizure outcome in univariable logistic regression analyses) resulted in the best model performance, while including all collected predictors led to a deterioration in model performance. Of interest, neither EEG nor semiology characteristics were predictive of seizure outcome in our univariable logistic regression analyses and were, therefore, not included in our models.

### 4.1 The illusory superiority of more complex models

There is a growing tendency to favor machine learning technology over traditional statistical modeling approaches when training models to predict postoperative seizure outcome. This is presumably due to an assumed superiority of highly sophisticated or complex models. As a result, a plethora of machine learning techniques have been deployed.^7^, ^8^, ^9^, ^10^, ^11^, ^12^, ^13^, ^14^, ^15^, ^16^, ^17^, ^18^, ^19^, ^20^, ^21^, ^22^, ^23^, ^24^, ^25^, ^26^, ^27^, ^28^, ^29^ It is, however, also increasingly recognized that the potential gains in predictive accuracy that have been attributed to more complex algorithms may have been inflated,^31^, ^36^ and that minor improvements observed “in the laboratory” may not translate into the real world.^31^

Previous studies that have used both machine learning techniques and traditional statistical modeling approaches to predict postoperative seizure outcome have found that logistic regression models perform as well as, or even better than, machine learning ones.^8^, ^9^, ^25^ To our knowledge, only one study by Yossofzai et al.^12^ has found that a machine learning model outperforms a logistic regression; however, this was a .01–.02 difference in AUC (.72 vs .73 in the train data set; .72 vs .74 in the test data set). This small improvement is unlikely to deliver an advantage in clinical practice. At the same time, using machine learning models introduces complexity, which in turn complicates their interpretation, implementation, and validation, and increases the risk of overfitting.

### 4.2 Larger samples mean higher accuracy… but only up until a certain point

There exists a general consensus in the machine learning community that more data, or larger sample sizes, equates to better model performance.^37^, ^38^ However, researchers have started to show that this is not always the case.^39^ We found that expanding our cohort beyond its current size (*N* = 797) nearly three‐fold did not provide meaningful gains.

Estimating the point of diminishing returns is invaluable because, although there is an abundance of unlabeled clinical data in our era of Big Data, (human) annotated clinical data remain scarce. Its creation is time‐consuming and requires the expertise of several clinical groups. Nevertheless, annotated data sets are essential in the creation of (supervised) learning algorithms. Generating learning curves can, therefore, inform researchers of the relative costs and benefits of adding additional annotated data to their model.^40^ Still, it is important to note that this learning curve is only an estimate and that actual model performance could exceed these predictions. Oversampling techniques that generate synthetic data could provide a data set that is similar in size to our expanded (predicted) data set; however, these approaches carry a risk of overfitting, as the synthetic data that they generate may closely resemble the original data set in a way that new data may not. The only way to validate this prediction is, therefore, to collect a sample size of several thousands of patients.

### 4.3 In pursuit of (geographical) model generalizability

Machine learning in clinical research is placing an increasing emphasis on model generalizability, where the highest level of evidence is achieved from applying models externally—to new centers. When we tested the model by Yossofzai et al.^12^ on our data, we found that it did not generalize well. This may at first glance seem surprising, as there is a striking similarity between our cohort and the cohort of Yossofzai et al.^12^—not only in terms of sample size but also in terms of patient characteristics and variables found to be predictive of seizure outcome. However, it also highlights a common issue related to the use of machine learning, namely, the tendency for models to overfit to local data. We, therefore, expect that a similar decrease in model performance would be demonstrated if another center were to use the machine learning models that we trained.

Different epilepsy surgery centers show variation in which diagnostic and therapeutic procedures are available, for which patients they are requested, and with which specifications they are carried out.^41^ Local practices also influence how data are annotated. Clinical data are interpreted by experts who assign a wide range of labels, from MRI diagnosis to epilepsy syndromes. Although official classification systems for annotation procedures exist,^42^, ^43^, ^44^, ^45^, ^46^, ^47^ individual studies often choose to—or are forced to—categorize their data ad hoc, primarily due to the restraints introduced by the retrospective nature of their data. Furthermore, not all experts will agree on the same label, which is evidenced by a lack of agreement regarding interpretation of EEG,^48^, ^49^, ^50^ MRI,^51^ positron emission tomography (PET),^51^ and histopathological data.^42^ It is thus possible that although our cohort and the cohort of Yossofzai et al.^12^ look similar on the surface, they may represent patients who have been characterized in a subtly different manner.

### 4.4 Limitations of the current study

The primary limitation of our study is that it is a retrospective study, which uses data originally obtained to understand patient disease and support clinical care, rather than to enable data analysis. These data are, therefore, at risk of being biased and incomplete.

#### 4.4.1 Biased data

Presurgical evaluation is largely standardized in that all patients undergo a full clinical history, structural MRI, and scalp‐ or video‐EEG, but the extent of further investigations will be patient dependent.^52^ To mitigate the occurrence of bias, we used a minimal data set, which included only clinical variables typically obtained for all epilepsy surgery patients. As such, we did not train our model using PET, single‐photon emission computed tomography (SPECT), magnetoencephalography (MEG), or functional MRI (fMRI) measures. One exception to this was the inclusion of genetic diagnosis, which we included despite not all patients having undergone genetic testing. The predictive value of genetic information in surgery candidate selection has not been systematically investigated.^53^ Consequently, we sought to contribute to this emerging area of research and provide initial evidence for its importance.

#### 4.4.2 Incomplete data

Related to the limitation of biased data is the limitation of incomplete data. Similar to past retrospective studies that have developed models for the prediction of seizure outcome after epilepsy surgery, we had a considerable amount of missing data. There are multiple ways of handling incomplete data sets, including deleting instances or replacing them with estimated values—a method known as imputation. Imputation techniques must, however, be used with caution, as they have limitations and can impact model performance.^54^ We, therefore, chose to drop instances where continuous data points were missing before including them into the model training data sets, and classified missing categorical data points as such, rather than using imputation.

### 4.5 Moving forward

Taken together, our findings suggest that (1) traditional statistical approaches such as logistic regression analyses are likely to perform as well as more complex machine learning models (when using routinely collected clinical predictors similar to those described here) and have advantages in interpretability, implementation, and generalizability; (2) collecting a large sample is important because it improves model performance and reduces overfitting, but including more than a thousand patients is unlikely to generate significant returns on data sets similar to ours; (3) model improvement is likely to come from data‐driven feature selection and exploring the inclusion of features that have thus far been overlooked or not undergone external validation due to barriers in study replication (discussed below).

Based on these findings, we make recommendations to advance our ability to predict seizure outcome after epilepsy surgery (Table 1). Surgery centers around the world must collaborate to produce high‐quality data for *research* purposes. Although models trained on single‐center data sets are likely to produce higher model performances than multi‐center data sets, they may not be suitable for use by other surgery centers. Critically, data must be collected and curated in a standardized manner, as highlighted by experts^55^ and similar to recent multi‐center endeavors.^9^, ^56^, ^57^ Here it will be important to distinguish between investigating variables that may be predictive of outcome and identifying variables that can (feasibly) be included as predictors in a clinical decision‐making tool. For the purpose of developing a clinical decision‐making tool, we suggest including only variables that are routinely collected for all epilepsy surgery patients at most centers, to avoid introducing bias into the model. In other words, researchers should carefully consider the added value of modalities such as MEG, PET, SPECT, and fMRI. It is notable that only variables obtained prior to surgery should be included in the model, as the aim is to create a predictive model. This means excluding variables such as postoperative measurement of resection and histopathology diagnosis. Reassuringly, we have shown that MRI diagnosis provides information similar to histopathology diagnosis. We also echo past recommendations^53^ in that we suggest avoiding variables that have repeatedly failed to predict outcome, as these have been shown to worsen model performance.

**TABLE 1:** Recommendations for future research.

1. Epilepsy surgery services should collaborate to create high‐quality data sets for research purposes
2. Data collection, annotation, and categorization should be standardized across surgery centers
3. Variables included as predictors in a clinical decision‐making tool should be limited to those that
(i) are routinely collected for all epilepsy surgery patients
(ii) can be obtained preoperatively, and
(iii) are significantly predictive of outcome
4. Data should be harmonized across surgery centers to tackle variability in data acquisition (e.g., variability induced by differences in MRI scanners and protocols)
5. Researchers should openly share their code on platforms (such as GitHub; https://github.com) to maximize transparency, support reproducibility, and enable external validation. In cases where code cannot be shared, researchers should share their models in a way that they can be validated by external centers

Training models using only clinical information is unlikely to procedure high model performance. Instead, better data must also entail new data. The inclusion of additional predictors to improve model performance may involve extracting quantitative features from preoperative MRI or EEG (as several studies have done^13^, ^14^, ^15^, ^16^, ^17^, ^18^, ^19^, ^20^, ^21^, ^22^, ^23^, ^24^, ^25^, ^26^, ^27^, ^28^, ^29^), characterizing the epileptogenic network through computational modeling,^58^ measuring lesion overlap with eloquent cortex,^59^ or adopting a network analysis approach.^60^ Here, it is important to note that machine learning techniques could provide superior performance compared to traditional statistical approaches if quantitative MRI and/or EEG features are used; however, to our knowledge, only one imaging study has to date compared these two approaches and found that they performed similarly well.^25^

It is important that all model software is made available—either as ready‐to‐use tools or openly shared code on platforms such as GitHub. Past studies have reported models capable of achieving accuracies of >90% using quantitative features extracted from MRI and EEG^14^, ^15^, ^19^, ^28^; however, none of these findings can be reproduced, and none of these models can be adopted by other centers, as there is insufficient information about how they were generated. Yossofzai et al.^12^ are to be commended for sharing their model in a way that allowed for it to be externally tested by ourselves and others.

## 5 CONCLUSIONS

Accurate prediction of seizure outcome after epilepsy surgery remains difficult. We highlight the importance of comparing traditional statistical modeling to complex machine learning techniques, as we show that these two approaches may perform equally well. We also demonstrate the importance of performing external validation of machine learning models, as we show that algorithms may underperform on other centers' data. Based on our findings, we present recommendations for future research, including the need for epilepsy services to collaborate in the creation of standardized data sets, the value of carefully choosing predictor variables for modeling, and the benefit of sharing models and code openly.

## AUTHOR CONTRIBUTIONS

MHE, TB, SA, and KW conceived and designed the study. MHE, JB, FM, KBD, CE, GC, MMT, PMS, RJP, APC, LM, and AM retrieved, anonymized, curated, and verified the data. MHE, MR, TB, SA, and KW analyzed the data, interpreted the results, and produced the figures. MHE wrote the manuscript. All authors edited and approved the final draft of the manuscript.

## FUNDING INFORMATION

This research is supported by the National Institute for Health Research Biomedical Research Centre at Great Ormond Street Hospital (NIHR GOSH BRC). The views expressed are those of the authors and not necessarily those of the NHS, the NIHR, or the Department of Health. The NIHR GOSH BRC had no role in the manuscript or the decision to submit it for publication. AC and RJP are supported by GOSH Children's Charity Surgeon Scientist Fellowships. SA is funded by the Rosetrees Trust (A2665). KW is supported by the Wellcome Trust (215901/Z/19/Z). MHE is supported by a Child Health Research Studentship, funded by NIHR GOSH BRC.

## CONFLICT OF INTEREST STATEMENT

JHC has acted as an investigator for studies with GW Pharmaceuticals, Zogenix, Vitaflo, Ovid, Marinius, and Stoke Therapeutics. She has been a speaker and on advisory boards for GW Pharmaceuticals, Zogenix, Biocodex, Stoke Therapeutics, and Nutricia; all remuneration has been paid to her department. She is president of the International League Against Epilepsy (2021–2025), and chair of the medical boards for Dravet UK, Hope 4 Hypothalamic Hamartoma, and Matthew's friends. MT has received grants from Royal Academy of Engineers and LifeArc. He has received honoraria from Medtronic. LM has received personal consultancy fees from Mendelian Ltd, outside the submitted work. AM has received honoraria from Biocodex and Nutricia, and provided consultancy to Biogen, outside the submitted work. All other authors report no disclosures relevant to the manuscript.

## STUDY APPROVAL STATEMENT

The study was approved by the National Research Ethics Service and registered with the Joint Research and Development Office of UCL Great Ormond Street Institute of Child Health and Great Ormond Street Hospital.

## PATIENT CONSENT STATEMENT

Informed patient consent for this retrospective assessment of our own clinical data was waived, provided that the data were handled anonymously by the clinical care team.

## Supporting information


Appendix S1
