# Supplementary material for: Predicting seizure outcome after epilepsy surgery: Do we need more complex models, larger samples, or better data?
Source: Epilepsia. 2023 Jun 16;64(8):2014–26. doi: 10.1111/epi.17637 (PMC10952307; doi:10.1111/epi.17637)
Supplement: Supplementary file 1 — Appendix S1 [file EPI-64-2014-s001.docx]

**Supplementary document accompanying the manuscript “Predicting seizure outcome after epilepsy surgery: do we need more complex models, larger samples, or better data?”**

Table of Contents

[Dataset description and data classification 2](#_Toc132720076)

[1. Demographic variables 2](#_Toc132720077)

[1.1. Handedness 2](#_Toc132720078)

[1.2. Educational status 2](#_Toc132720079)

[2. Epilepsy characteristics 2](#_Toc132720080)

[2.1. Age at first seizure 2](#_Toc132720081)

[2.2. Age of epilepsy onset 2](#_Toc132720082)

[2.3. Febrile seizures 2](#_Toc132720083)

[2.4. Family history of epilepsy 2](#_Toc132720084)

[2.5. Pre-operative antiseizure medication 2](#_Toc132720085)

[2.6. Age at surgery 3](#_Toc132720086)

[2.7. Duration of epilepsy 3](#_Toc132720087)

[3. Pre-operative seizure classification 3](#_Toc132720088)

[3.1. Seizure history 3](#_Toc132720089)

[3.2. Current seizures 3](#_Toc132720090)

[3.3. Seizure semiology 3](#_Toc132720091)

[3.4. Ictal and interictal EEG recordings 3](#_Toc132720092)

[4. Pre-operative MRI findings 4](#_Toc132720093)

[4.1. MRI laterality 4](#_Toc132720094)

[4.2. MRI diagnosis 4](#_Toc132720095)

[5. Genetic results 4](#_Toc132720096)

[6. Surgery details 5](#_Toc132720097)

[6.1. Type of procedure performed 5](#_Toc132720098)

[6.2. Side operated on 5](#_Toc132720099)

[6.3. Lobe operated on 5](#_Toc132720100)

[7. Histopathology diagnoses 5](#_Toc132720101)

[Supplementary Tables 7](#_Toc132720102)

[Supplementary Figures 18](#_Toc132720103)

# Dataset description and data classification

## Demographic variables

### Handedness

Patients’ (pre-operative) handedness was coded as one of five possible groups: Left, Right, Ambidextrous, or No clear hand preference.

### Educational status

Patients’ (pre-operative) educational status was coded as one of six possible groups: Mainstream school, Mainstream school with additional support, Special educational needs school, Home schooled, Residential home, or Not of school age.

## Epilepsy characteristics

### Age at first seizure

Age at first seizure was a continuous variable used to capture the age at which the patient experienced their first seizure. This could have been an afebrile or febrile seizure.

### Age of epilepsy onset

Age of epilepsy onset was a continuous variable used to capture the age at which the patient was diagnosed with epilepsy. The variables “Age at first seizure” and ”Age of epilepsy onset” were kept distinct to account for early, isolated occurrences of febrile seizure(s), which did not result in a diagnosis of epilepsy. For example, if the child experienced a single febrile seizure at 7 months and was subsequently seizure-free until the age of 5 years, at which time they experienced afebrile seizure(s), the child’s age at first seizure would have been classified as 7 months, while their age of epilepsy onset would have been classified as 5 years. However, if the child experienced afebrile seizures immediately following their febrile seizure at 7 months, their age at first seizure and age of epilepsy onset would have been the same.

### Febrile seizures

Patients were classified as either having or not having a history of febrile seizures.

### Family history of epilepsy

Patients were classified as either having or not having a family history of epilepsy. No criteria were applied in terms of how closely related the affected family member(s) needed to be.

### Pre-operative antiseizure medication

Patients’ antiseizure medication was characterized in terms of 1) the number of antiseizure medications the patient was receiving at time of pre-surgical evaluation, and 2) the total number of different antiseizure medications the patient had trialed from epilepsy onset to pre-surgical evaluation.

### Age at surgery

Age at surgery was a continuous variable used to capture the age at which the patient underwent their first resective or disconnective surgery for epilepsy.

### Duration of epilepsy

Duration of epilepsy was a continuous variable used to capture the time elapsed between the patient’s epilepsy onset and surgery (“Age at surgery” - “Age of epilepsy onset” = “Duration of epilepsy”).

## Pre-operative seizure classification

Patients’ seizure history as well as their seizures, seizure semiology, and EEG recordings at the time of pre-surgical evaluation were retrospectively characterized by four experienced pediatric neurophysiology consultants: FM, KD, CE and GC. The consultants had access only to the patients’ pre-operative information (i.e. they did not have access to the patients’ seizure outcomes).

### Seizure history

The consultants coded whether patients had a history of: Infantile seizures, Electrical status epilepticus during slow-wave sleep (ESES), Status epilepticus, and Generalized tonic-clonic seizures.

### Current seizures

The consultants coded the patients’ seizures at the time of pre-surgical evaluation: Spasms, Number of seizure types, Focal versus generalized onset (all focal/all generalized/mixture of focal and generalized), Motor versus non-motor (all motor/all non-motor/mixture of motor and non-motor), and Aware versus impaired awareness (all aware/all impaired awareness/mixture of aware and impaired awareness).

### Seizure semiology

The consultants coded the patients’ seizure semiology at the time of pre-surgical evaluation: Localizing + lesion concordant, Lateralizing + lesion concordant, and Non-concordant.

### Ictal and interictal EEG recordings

The consultants coded the patients’ ictal and interictal EEG recordings at the time of pre-surgical evaluation: Localizing + lesion concordant, Lateralizing + lesion concordant, and Non-concordant.

## Pre-operative MRI findings

### MRI bilaterality

Patients’ pre-operative MRI scan was classified as either abnormal (MRI positive) or normal (MRI negative). MRI scans classified as abnormal were further classified as comprising a unilateral (right- or left-sided) abnormality or a bilateral abnormality.

### MRI diagnosis

In addition to MRI bilaterality, MRI findings were characterized as one of fifteen possible diagnoses.

| **MRI diagnoses.** |
| --- |
| (1) FCD-II (focal cortical dysplasia type II) |
| (2) FCD-NOS (focal cortical dysplasia not otherwise specified) |
| (3) LEAT (low-grade epilepsy-associated tumor, including ganglioglioma, dysembryoplastic neuroepithelial tumor, pilocytic astrocytoma, and papillary glioneuronal tumor) |
| (4) LGL (low-grade lesion not otherwise specified, comprising possible diagnoses focal cortical dysplasia, mesial temporal sclerosis, and low-grade epilepsy-associated tumor) |
| (5) MCD-Other (including hypothalamic hamartoma, hemimegalencephaly, polymicrogyria, and focal cortical dysplasia type III) |
| (6) MTS (mesial temporal sclerosis, including hippocampal sclerosis, neuronal loss in the hippocampus/temporal lobe, gliosis in hippocampus/temporal lobe, granule cell dispersion, and Chaslin’s gliosis) |
| (7) Negative |
| (8) N-LEAT (non-low-grade epilepsy-associated tumor, including astrocytoma, neurocytoma, and pleomorphic xanthoastrocytoma) |
| (9) NSC (non-specific epilepsy-associated changes) |
| (10) Not determined lesion/abnormality |
| (11) Rasmussen encephalitis |
| (12) Scarring (as result of an acute vascular event, i.e. hemorrhagic infarction, trauma, or infection in the past), |
| (13) TS (tuberous sclerosis) |
| (14) Unspecified tumor |
| (15) Vascular diagnoses (including cavernoma, meningioangiomatosis, as seen in Sturge-Weber syndrome, and arterio-venous malformations) |

## 5. Genetic results

All genetic tests performed in children who underwent surgery were retrospectively retrieved. The reported genetic variants were reviewed by APC, an experienced clinical scientist with a specialization in molecular genetics. Variant classification was carried out using Alamut Visual version 2.15 (SOPHiA GENETICS, Lausanne, Switzerland). Population data (<https://gnomad.broadinstitute.org/>), *in silico* tools predictions, functional analysis, segregation studies, and previous reports were taken into account. Variants were classified using the American College of Medical Genetics (ACMG)/Association for Molecular Pathology (AMP) guidelines (Richards et al., 2015) and Association for Clinical Genomic Science (ACGS) Best Practice Guidelines for Variant Classification in Rare Disease 2020 (<https://www.acgs.uk.com/quality/best-practice-guidelines/>).

Findings were discussed with LM, a clinical geneticist, and AM, a consultant pediatric neurologist. Variants were classified into five categories as per standard ACMG guidelines, namely: 1) benign, 2) likely benign, 3) uncertain significance, 4) likely pathogenic, and 5) pathogenic. We considered patients with variants classified as pathogenic, likely pathogenic and variants of uncertain significance with a suspicion of being pathogenic to have a genetic cause of epilepsy, in accordance with ACGS Best Practice Guidelines for Variant Classification in Rare Disease 2020 (<https://www.acgs.uk.com/quality/best-practice-guidelines/>). The clinicians did not have access to the patients’ seizure outcomes during classification of genetic information.

## 6. Surgery details

Surgeries were classified by visual inspection of pre- and post-operative MRI scans and retrieval of medical records describing the pre-operative intent, surgical procedure, and post-operative discharge. Surgeries were characterized according to: Type of procedure performed, Side operated on, and Lobe operated on.

### 6.1. Type of procedure performed

Type of procedure performed included four categories: Lesionectomy, Lobectomy, Disconnection and Hemispherotomy. Procedures comprising more than one type of procedure, so-called “Combined procedures”, were reported as such, but re-classified for modelling purposes. For re-classification, the more extensive procedure was used. For example, a surgery comprising both a Lobectomy and Lesionectomy would be re-classified as a Lobectomy.

### Side operated on

Side operated on included two categories: Right and Left. When classifying Side operated on, “Not applicable” was assigned to focal resections that involved the removal of a hypothalamic hamartoma.

### Lobe operated on

Lobe operated on included six categories: Temporal, Frontal, Parietal, Occipital, Insular and Multilobar. When classifying Lobe operated on, “Not applicable” was assigned to hemispherotomy procedures as well as focal resections that involved the removal of a hypothalamic hamartoma.

## Histopathology diagnoses

Histopathology diagnosis was grouped into one of thirteen categories.

| **Histopathology diagnoses.** |
| --- |
| (1) FCD-II (focal cortical dysplasia type II) |
| (2) FCD-NOS (focal cortical dysplasia not otherwise specified) |
| (3) LEAT (low-grade epilepsy-associated tumor, including ganglioglioma, dysembryoplastic neuroepithelial tumor, pilocytic astrocytoma, and papillary glioneuronal tumor) |
| (4) M-MCD (mild malformation of cortical development, including focal cortical dysplasia type I) |
| (5) MCD-Other (including hypothalamic hamartoma, hemimegalencephaly, polymicrogyria, and focal cortical dysplasia type III) |
| (6) MTS (mesial temporal sclerosis, including hippocampal sclerosis, neuronal loss in the hippocampus/temporal lobe, gliosis in hippocampus/temporal lobe, granule cell dispersion, and Chaslin’s gliosis) |
| (7) N-LEAT (non-low-grade epilepsy-associated tumor, including astrocytoma, neurocytoma, and pleomorphic xanthoastrocytoma) |
| (8) Normal result |
| (9) NSC (non-specific epilepsy-associated changes) |
| (10) Rasmussen encephalitis |
| (11) Scarring (as result of an acute vascular event, i.e. hemorrhagic infarction, trauma, or infection in the past), |
| (12) TS (tuberous sclerosis) |
| (13) Vascular diagnoses (including cavernoma, meningioangiomatosis, as seen in Sturge-Weber syndrome, and arterio-venous malformations) |

# Supplementary Tables

| **Supplementary Table 1. Demographic information and clinical characteristics (N = 797).** | |
| --- | --- |
| **Sex** N (% of total sample) |  |
| Males | 409 (51) |
| Females | 386 (48) |
| Missing data | 2 (<1) |
| **Handedness** N (% of total sample) |  |
| Right | 470 (59) |
| Left | 196 (25) |
| No clear hand preference/Ambidextrous | 57 (7) |
| Missing data | 74 (9) |
| **Educational status** |  |
| Mainstream | 202 (25) |
| Mainstream with support | 182 (23) |
| SEN school | 137 (17) |
| Home-schooled/Residential home | 15 (2) |
| Not of school age | 215 (27) |
| Missing data | 46 (6) |
| **Epilepsy characteristics** years, median [IQR] (range) |  |
| Age at first seizure*^a^* | 1.3 [0.3-4.0] (0-15.6) |
| Age of epilepsy onset | 1.7 [0.3-5.0] (0-15.6) |
| Age at surgery | 8.4 [4.4-13.3] (0.1-21.5) |
| Duration of epilepsy | 4.9 [2.4-8.4] (0-20.7) |
| **Antiseizure medication (ASM)** N (% of total sample) |  |
| **Number of ASM at pre-operative evaluation** |  |
| 0 | 8 (1) |
| 1 | 137 (17) |
| 2 | 318 (39) |
| 3 | 224 (28) |
| 4+ | 79 (10) |
| Missing data | 31 (4) |
| **Total number of ASM trialed (from epilepsy onset to surgery)** |  |
| 0 | 2 (<1) |
| 1-2 | 106 (13) |
| 3-4 | 270 (34) |
| 5-6 | 210 (26) |
| 7-8 | 110 (14) |
| 9+ | 53 (7) |
| Missing data | 46 (6) |
| **Pre-operative MRI findings** N (% of total sample) |  |
| **MRI laterality** |  |
| Left | 372 (47) |
| Right | 315 (40) |
| Bilateral | 92 (12) |
| Negative | 12 (2) |
| Not applicable | 1 (<1) |
| Missing data | 5 (<1) |
| **MRI status** |  |
| Focal | 451 (57) |
| Diffuse | 241 (30) |
| Multifocal | 88 (11) |
| Negative | 12 (2) |
| Missing data | 5 (<1) |
| **MRI diagnosis** |  |
| LEAT | 118 (15) |
| FCD-II | 118 (15) |
| Scarring | 107 (13) |
| MCD-Other | 95 (12) |
| MTS | 74 (9) |
| Not determined lesion/abnormality | 61 (8) |
| FCD-NOS | 47 (6) |
| Tuberous sclerosis | 45 (6) |
| Vascular | 40 (5) |
| Rasmussen encephalitis | 39 (5) |
| Low grade lesion | 16 (2) |
| Negative | 12 (2) |
| Unspecified tumor | 9 (1) |
| N-LEAT | 6 (<1) |
| NSC | 5 (<1) |
| Missing data | 5 (<1) |
| **Genetic findings** N (% of total sample) |  |
| Pathogenic/likely pathogenic SNV | 19 (2) |
| CNV | 9 (1) |
| Benign/likely benign SNV | 26 (3) |
| No variant identified | 53 (7) |
| No test administered | 690 (87) |
| **Surgery information** N (% of total sample) |  |
| **Type of surgery** N (% of total sample) |  |
| Lesionectomy | 317 (40) |
| Lobectomy | 219 (27) |
| Hemispherotomy | 204 (26) |
| Disconnection | 57 (7) |
| **Side operated on** N (% of total sample) |  |
| Left | 416 (52) |
| Right | 377 (47) |
| Not applicable*^b^* | 4 (<1) |
| **Lobe operated on** N (% of total sample) |  |
| Temporal | 278 (35) |
| Frontal | 152 (19) |
| Multilobar | 85 (11) |
| Parietal | 47 (6) |
| Occipital | 15 (2) |
| Insular | 2 (<1) |
| Not applicable*^c^* | 218 (27) |
| **Histopathology** N (% of total sample) |  |
| LEAT | 153 (19) |
| FCD-II | 119 (15) |
| MTS | 72 (9) |
| Scarring | 67 (8) |
| MCD-Other | 62 (8) |
| NSC | 49 (6) |
| Tuberous sclerosis | 44 (6) |
| Vascular | 32 (4) |
| Rasmussen encephalitis | 27 (3) |
| Normal result | 19 (2) |
| N-LEAT | 13 (2) |
| FCD-NOS | 9 (1) |
| M-MCD | 6 (<1) |
| Histopathology not collected or report not available | 125 (16) |
|  | |
| *^a^* Age at first seizure and Age of epilepsy onset were kept distinct to account for early, isolated occurrences of febrile seizures (see Supplementary Material p. 2).  *^b^* Not applicable was assigned to focal resections that involved the removal of a hypothalamic hamartoma.  *^c^* Not applicable was assigned to hemispherotomy as well as focal resections that involved the removal of a hypothalamic hamartoma. | |
| Abbreviations: ASM = Antiseizure medication; CNV = Copy number variation; DNET = Dysembryoplastic neuroepithelial tumor; FCD = Focal cortical dysplasia; FCD-NOS = Focal cortical dysplasia not otherwise specified; IQR = Interquartile range; LEAT = Low-grade epilepsy-associated tumor; MCD-Other = Malformation of cortical development-other; M-MCD = Mild malformation of cortical development; MTS = Mesial temporal sclerosis; NA = Not applicable; N-LEAT = Non-low-grade epilepsy-associated tumor; NSC = Non-specific epilepsy-associated changes; SEN = Special educational needs school; SNV = Single nucleotide variation. | |

| **Supplementary Table 2. Past and current seizure information (N = 797).** | |
| --- | --- |
| **Family history of epilepsy** |  |
| Yes | 215 (27) |
| No | 580 (73) |
| Missing data | 2 (<1) |
| **Seizure history** N (% of total sample) |  |
| **History of infantile spasms** |  |
| Yes | 145 (18) |
| No | 593 (74) |
| Missing data | 59 (7) |
| **History of febrile seizures** |  |
| Yes | 113 (14) |
| No | 669 (84) |
| Missing data | 15 (2) |
| **History of ESES** |  |
| Yes | 37 (5) |
| No | 679 (85) |
| Missing data | 81 (10) |
| **History of status epilepticus** |  |
| EPC | 22 (3) |
| Non-convulsive | 12 (2) |
| Convulsive | 103 (13) |
| Mixed | 13 (2) |
| None | 588 (74) |
| Missing data | 59 (7) |
| **History of generalized tonic-clonic seizures** |  |
| Yes | 319 (40) |
| No | 414 (52) |
| Missing data | 64 (8) |
| **Seizures at time of pre-operative evaluation** N (% of total sample) |  |
| **Spasms** |  |
| Yes | 148 (19) |
| No | 599 (75) |
| Missing data | 50 (6) |
| **Number of seizure types** |  |
| 0 | 10 (1) |
| 1 | 357 (45) |
| 2 | 239 (30) |
| 3 | 97 (12) |
| 4 | 29 (4) |
| 5 | 4 (<1) |
| 6 | 2 (<1) |
| Missing data | 59 (7) |
| **Seizure types** |  |
| *Focal vs. generalized onset* |  |
| All focal | 566 (71) |
| All generalized | 3 (<1) |
| Mixed focal and generalized | 109 (14) |
| Missing data | 119 (15) |
| *Motor vs. non-motor onset* |  |
| All motor | 340 (43) |
| All non-motor | 84 (11) |
| Mixed motor and non-motor | 309 (39) |
| Missing data | 64 (8) |
| *Aware vs. impaired awareness onset* |  |
| All aware | 55 (7) |
| All impaired awareness | 334 (42) |
| Mixed aware and impaired awareness | 185 (23) |
| Missing data | 223 (28) |
| **Seizure semiology** |  |
| Localizing | 414 (52) |
| Lateralizing | 113 (14) |
| Non-concordant | 159 (20) |
| Missing data | 111 (14) |
| **EEG** |  |
| *Ictal EEG* |  |
| Localizing | 354 (44) |
| Lateralizing | 113 (14) |
| Non-concordant | 136 (17) |
| Missing data | 194 (24) |
| *Interictal EEG* |  |
| Localizing | 428 (54) |
| Lateralizing | 129 (16) |
| Non-concordant | 144 (18) |
| Missing data | 96 (12) |
|  | |
| Abbreviations: EEG = Electroencephalography; EPC = Epilepsia partialis continua; ESES = Electrical status epilepticus during slow-wave sleep. | |

| **Supplementary Table 3. Pathogenic and likely pathogenic single nucleotide variants (SNVs) and their associated pre-operative MRI findings, surgery types, histopathology diagnoses, and post-operative seizure outcomes.** | | | | | | | | | | |
| --- | --- | --- | --- | --- | --- | --- | --- | --- | --- | --- |
| **Variant** | **Gene function** | **Inheritance** | **Classification** | **ACMG criteria** | **MRI findings** | **Procedure** | **Histopathology** | **Time of test** | **Outcome** |  |
| *DEPDC5*  c.280-10T>G | mTOR pathway regulator | Unknown | Uncertain clinical significance – warm 3 | PM2_Mod; PP3 | Focal cortical dysplasia | Lesionectomy | Not available | After surgery | N-SF |  |
| *GRIN2B*  c.2453T>C; p.(Met818Thr) | Glutamate receptor component | *De novo* | Likely pathogenic | PM2_Mod; PP3; PP2; PM6_Mod; PS4_Sup | Polymicrogyria | Hemispherotomy | Polymicrogyria | After surgery | N-SF |  |
| *KRIT1*  c.1878dupA, p.(Gln627Thrfs*28) | Microtubule associated protein associated with formation of cerebral cavernous malformations | *De novo* | Pathogenic | PVS1_Very strong; PM2_Mod; PM6_Sup | Cavernoma | Lesionectomy | Cavernoma | After surgery | NR |  |
| *KRIT1*  c.2043del p.(Lys682Serfs*25) | Microtubule associated protein associated with formation of cerebral cavernous malformations | Strong family history | Likely pathogenic | PVS1_Strong + PM2_Mod | Cavernoma | Lobectomy | Cavernoma | Results known at pre-surgical evaluation | SF |  |
| *NSD1*  c.1237-6T>G het | Histone methyltransferase | Unknown | Uncertain clinical significance – warm 3 | PM2_Mod; PP3 | Undetermined lesion | Lobectomy | DNET | After surgery | N-SF |  |
| *SCN1A*  c.4888G>A (p.Val1630Met) | Sodium channel component | *De novo* | Likely pathogenic | PM2_Mod; PM5_Mod; PM6_Sup; PP3; PP2 | Hippocampal sclerosis | Lobectomy | Hippocampal sclerosis | Results known at pre-surgical evaluation | SF |  |
| *SCN1A*  c.652T>C p.(Phe218Leu) | Sodium channel component | Maternally inherited (one sibling also has the variant; unaffected apart from single febrile seizure) | Likely pathogenic | PM2_Mod; PP3; PP2; PS4_Mod | Hippocampal sclerosis | Lobectomy | Hippocampal sclerosis | Results known at pre-surgical evaluation | N-SF |  |
| *SCN2A*  c.4841T>C p.(Leu1614Pro) | Sodium channel component | *De novo* | Likely pathogenic | PM2_Mod; PP3; PP2; PS4_Sup; PM6_Sup | Polymicrogyria + Cortical dysplasia | Disconnection + Lobectomy | Polymicrogyria + Hippocampal sclerosis | After surgery | N-SF |  |
| *TSC1*  c.2283C>G p.(Tyr761*) | mTOR pathway regulator | Paternally inherited (family history of TSC) | Pathogenic | PVS1_Very strong; PM2_Mod; PS4_Mod | Tuberous sclerosis | Lesionectomy | Tuberous sclerosis | Results known at pre-surgical evaluation | N-SF |  |
| *TSC1*  c.2593C<T (p.Gln865*) | mTOR pathway regulator | Paternally inherited | Pathogenic | PVS1_Very strong; PM2_Mod | Tuberous sclerosis | Lesionectomy | Tuberous sclerosis | Results known at pre-surgical evaluation | SF |  |
| *TSC1 ^a^* | mTOR pathway regulator | Father has a history of epilepsy and clinical signs suggestive of TSC | Variant information missing | Variant information missing | Tuberous sclerosis | Lobectomy | Tuberous sclerosis | Results known at pre-surgical evaluation | N-SF |  |
| *TSC2*  c.2251C>T (p.Arg751*) | mTOR pathway regulator | Unknown | Pathogenic | PVS1_Very strong: PM2_Mod; PS4_Mod | Tuberous sclerosis | Lobectomy | Tuberous sclerosis | Results known at pre-surgical evaluation | N-SF |  |
| *TSC2*  c.2713C>T p.(Arg905Trp) | mTOR pathway regulator | *De novo* | Pathogenic | PM2_Mod; PS4_Mod; PM6_Strong; PM5_Mod; PP3 | Tuberous sclerosis | Lesionectomy | Tuberous sclerosis | Results known at pre-surgical evaluation | SF |  |
| *TSC2*  c.4006-1G>A | mTOR pathway regulator | Inherited from an affected father | Pathogenic | PVS1_Very strong: PM2_Mod | Focal cortical dysplasia | Lobectomy | Focal cortical dysplasia type II | Results known at pre-surgical evaluation | SF |  |
| *TSC2*  c.4927A>C p.(Asn1643His) | mTOR pathway regulator | Unknown | Likely pathogenic | PM2_Mod; PS4_Mod; PM5_Mod; PP3 | Tuberous sclerosis | Lesionectomy | Tuberous sclerosis | Results known at pre-surgical evaluation | N-SF |  |
| *TSC2* deletion of exon 32 | mTOR pathway regulator | Unknown | Uncertain clinical significance | PVS1_Mod; PM2_Mod | Tuberous sclerosis | Lesionectomy | Tuberous sclerosis | Results known at pre-surgical evaluation | NR |  |
| *TSC2 ^a^* | mTOR pathway regulator | *De novo* | Variant information missing | Variant information missing | Tuberous sclerosis + SEGA | Lesionectomy | SEGA | After surgery | N-SF |  |
| *TSC2 ^a^* | mTOR pathway regulator | Variant information missing | Variant information missing | Variant information missing | Tuberous sclerosis + SEGA | Lesionectomy | Tuberous sclerosis | Results known at pre-surgical evaluation | N-SF |  |
| *TSC2 ^a^* | mTOR pathway regulator | *De novo* | Variant information missing | Variant information missing | Tuberous sclerosis | Lesionectomy | Tuberous sclerosis | After surgery | N-SF |  |
|  | | | | | | | | | | |
| ACGM = The American College of Medical Genetics and Genomics; NR = Not reported; N-SF = Not seizure-free; SEGA = Subependymal giant cell astrocytoma; SF = Seizure-free; TSC = Tuberous sclerosis complex.  *^a^*  Variant information not available. | | | | | | | | | | |

| **Supplementary Table 4. Pathogenic and likely pathogenic copy number variations (CNVs) and their associated pre-operative MRI findings, surgery types, histopathology diagnoses, and post-operative seizure outcomes.** | | | | | | | | |
| --- | --- | --- | --- | --- | --- | --- | --- | --- |
| **Variant** | **Inheritance** | **Classification** | **MRI findings** | **Procedure** | **Histopathology** | **Time of test** | **Outcome** |  |
| Ring chromosome 22 | Presumed not inherited as parents healthy | Specific details of abnormality not available. May be contributing to phenotype. | Undetermined abnormality | Abandoned | Not collected | Results known at pre-surgical evaluation | NA |  |
| Chromosome 15  microdeletion 15q13.2-15q13.3 | Maternally inherited | Array finding consistent with phenotype. | Cortical dysplasia | Disconnection + Lesionectomy | Non-specific epilepsy-associated changes | Results known at pre-surgical evaluation | N-SF |  |
| Chromosome 2 copy number loss 2q14.3 | Paternally inherited | May be contributing to phenotype. | Infarct | Hemispherotomy | Hippocampal sclerosis | Results known at pre-surgical evaluation | SF |  |
| Chromosome 5 copy number loss involving *MCC2* gene | Maternally inherited | Specific details of abnormality not available. May be contributing to phenotype. | Focal cortical dysplasia | Lesionectomy | Focal cortical dysplasia type II | Results known at pre-surgical evaluation | N-SF |  |
| Chromosome 12 copy number gain | Maternally inherited | Specific details of abnormality not available. Contribution to phenotype unclear. | Cortical malformation | Lesionectomy | Polymicrogyria | After surgery | NR |  |
| Chromosome 1 copy number gain 1q22-1q22.3 | Inheritance unknown | Specific details of abnormality not available. Contribution to phenotype unclear. | Focal cortical dysplasia | Lesionectomy | Focal cortical dysplasia type II | Results known at pre-surgical evaluation | N-SF |  |
| Mosaic trisomy 13 | Not inherited as mosaic | Specific details of abnormality not available. May be contributing to phenotype. | Focal cortical dysplasia | Lobectomy | Hippocampal sclerosis | Results known at pre-surgical evaluation | SF |  |
| Chromosome 7 copy number gain 7q11.23 including *ELN* and *LIMK1* genes | Not present in mother, paternal status unknown | Contribution to phenotype unclear. | Hemimegalencephaly | Hemispherotomy | Hemimegalencephaly | After surgery | N-SF |  |
| Chromosome 2 copy number loss  2p24.3 including *MYCN* gene | Unknown | Contribution to phenotype unclear. | Infarct | Hemispherotomy | Not available | After surgery | SF |  |
|  | | | | | | | | |
| Abbreviations: NR = Not reported; N-SF = Not seizure-free; SF = Seizure-free. | | | | | | | | |

| **Supplementary Table 5. Relationships between demographic, clinical and surgical variables. Pairwise comparison following correction for multiple comparison using the Holm method.** | | | | |
| --- | --- | --- | --- | --- |
| **Variable 1** | **Variable 2** | ***P* value** | **Sig.** | **Sig. corr.** |
| Sex | Handedness | 8.69E-01 | ns | ns |
| Sex | Educational status | 1.13E-01 | ns | ns |
| Sex | Family history | 3.28E-01 | ns | ns |
| Sex | Genetic findings | 9.63E-01 | ns | ns |
| Sex | Age of epilepsy onset | 8.50E-01 | ns | ns |
| Sex | Age at surgery | 8.56E-01 | ns | ns |
| Sex | Duration of epilepsy | 9.78E-01 | ns | ns |
| Sex | ASM pre-op | 8.96E-01 | ns | ns |
| Sex | ASM trialed | 9.55E-01 | ns | ns |
| Sex | Pre-op MRI side | 6.61E-01 | ns | ns |
| Sex | Pre-op MRI diagnosis | 6.52E-04 | sig | sig |
| Sex | Procedure type | 5.81E-01 | ns | ns |
| Sex | Hemisphere operated on | 7.78E-01 | ns | ns |
| Sex | Lobe operated on | 6.40E-01 | ns | ns |
| Sex | Histopathology diagnosis | 6.57E-02 | ns | ns |
| Sex | Seizure outcome | 1.00E+00 | ns | ns |
| Handedness | Educational status | 4.21E-10 | sig | sig |
| Handedness | Family history | 1.39E-01 | ns | ns |
| Handedness | Genetic findings | 4.96E-01 | ns | ns |
| Handedness | Age of epilepsy onset | 4.74E-15 | sig | sig |
| Handedness | Age at surgery | 2.20E-16 | sig | sig |
| Handedness | Duration of epilepsy | 4.67E-11 | sig | sig |
| Handedness | ASM pre-op | 2.13E-01 | ns | ns |
| Handedness | ASM trialed | 5.20E-03 | sig | ns |
| Handedness | Pre-op MRI side | 2.20E-16 | sig | sig |
| Handedness | Pre-op MRI diagnosis | 1.84E-08 | sig | sig |
| Handedness | Procedure type | 3.57E-03 | sig | ns |
| Handedness | Hemisphere operated on | 2.20E-16 | sig | sig |
| Handedness | Lobe operated on | 5.12E-03 | sig | ns |
| Handedness | Histopathology diagnosis | 4.39E-08 | sig | sig |
| Handedness | Seizure outcome | 7.92E-03 | sig | ns |
| Educational status | Family history | 2.71E-01 | ns | ns |
| Educational status | Genetic findings | 4.13E-03 | sig | ns |
| Educational status | Age of epilepsy onset | 2.20E-16 | sig | sig |
| Educational status | Age at surgery | 2.20E-16 | sig | sig |
| Educational status | Duration of epilepsy | 2.20E-16 | sig | sig |
| Educational status | ASM pre-op | 2.07E-06 | sig | sig |
| Educational status | ASM trialed | 7.73E-13 | sig | sig |
| Educational status | Pre-op MRI side | 9.96E-01 | ns | ns |
| Educational status | Pre-op MRI diagnosis | 3.42E-15 | sig | sig |
| Educational status | Procedure type | 4.45E-12 | sig | sig |
| Educational status | Hemisphere operated on | 7.19E-01 | ns | ns |
| Educational status | Lobe operated on | 1.25E-14 | sig | sig |
| Educational status | Histopathology diagnosis | 6.58E-08 | sig | sig |
| Educational status | Seizure outcome | 3.74E-05 | sig | sig |
| Family history | Genetic findings | 5.37E-03 | sig | ns |
| Family history | Age of epilepsy onset | 4.64E-01 | ns | ns |
| Family history | Age at surgery | 6.11E-02 | ns | ns |
| Family history | Duration of epilepsy | 1.66E-02 | sig | ns |
| Family history | ASM pre-op | 2.90E-01 | ns | ns |
| Family history | ASM trialed | 6.37E-01 | ns | ns |
| Family history | Pre-op MRI side | 9.07E-01 | ns | ns |
| Family history | Pre-op MRI diagnosis | 1.16E-01 | ns | ns |
| Family history | Procedure type | 1.27E-02 | sig | ns |
| Family history | Hemisphere operated on | 1.00E+00 | ns | ns |
| Family history | Lobe operated on | 8.28E-03 | sig | ns |
| Family history | Histopathology diagnosis | 1.03E-02 | sig | ns |
| Family history | Seizure outcome | 9.81E-01 | ns | ns |
| Genetic findings | Age of epilepsy onset | 2.81E-02 | sig | ns |
| Genetic findings | Age at surgery | 2.14E-02 | sig | ns |
| Genetic findings | Duration of epilepsy | 4.88E-01 | ns | ns |
| Genetic findings | ASM pre-op | 3.16E-01 | ns | ns |
| Genetic findings | ASM trialed | 1.49E-01 | ns | ns |
| Genetic findings | Pre-op MRI side | 8.79E-02 | ns | ns |
| Genetic findings | Pre-op MRI diagnosis | 1.74E-10 | sig | sig |
| Genetic findings | Procedure type | 2.27E-01 | ns | ns |
| Genetic findings | Hemisphere operated on | 7.69E-01 | ns | ns |
| Genetic findings | Lobe operated on | 8.01E-01 | ns | ns |
| Genetic findings | Histopathology diagnosis | 8.13E-12 | sig | sig |
| Genetic findings | Seizure outcome | 2.00E-05 | sig | sig |
| Age of epilepsy onset | Age at surgery | 2.20E-16 | sig | sig |
| Age of epilepsy onset | Duration of epilepsy | 5.85E-04 | sig | sig |
| Age of epilepsy onset | ASM pre-op | 1.32E-05 | sig | sig |
| Age of epilepsy onset | ASM trialed | 1.49E-06 | sig | sig |
| Age of epilepsy onset | Pre-op MRI side | 7.61E-01 | ns | ns |
| Age of epilepsy onset | Pre-op MRI diagnosis | 2.20E-16 | sig | sig |
| Age of epilepsy onset | Procedure type | 9.38E-12 | sig | sig |
| Age of epilepsy onset | Hemisphere operated on | 2.85E-01 | ns | ns |
| Age of epilepsy onset | Lobe operated on | 2.20E-16 | sig | sig |
| Age of epilepsy onset | Histopathology diagnosis | 2.20E-16 | sig | sig |
| Age of epilepsy onset | Seizure outcome | 1.15E-04 | sig | sig |
| Age at surgery | Duration of epilepsy | 2.20E-16 | sig | sig |
| Age at surgery | ASM pre-op | 1.95E-04 | sig | sig |
| Age at surgery | ASM trialed | 2.01E-01 | ns | ns |
| Age at surgery | Pre-op MRI side | 6.37E-01 | ns | ns |
| Age at surgery | Pre-op MRI diagnosis | 2.20E-16 | sig | sig |
| Age at surgery | Procedure type | 7.34E-12 | sig | sig |
| Age at surgery | Hemisphere operated on | 1.53E-01 | ns | ns |
| Age at surgery | Lobe operated on | 1.38E-14 | sig | sig |
| Age at surgery | Histopathology diagnosis | 3.23E-10 | sig | sig |
| Age at surgery | Seizure outcome | 2.76E-01 | ns | ns |
| Duration of epilepsy | ASM pre-op | 7.77E-02 | ns | ns |
| Duration of epilepsy | ASM trialed | 1.43E-10 | sig | sig |
| Duration of epilepsy | Pre-op MRI side | 8.99E-01 | ns | ns |
| Duration of epilepsy | Pre-op MRI diagnosis | 2.20E-16 | sig | sig |
| Duration of epilepsy | Procedure type | 5.25E-08 | sig | sig |
| Duration of epilepsy | Hemisphere operated on | 6.96E-01 | ns | ns |
| Duration of epilepsy | Lobe operated on | 4.46E-05 | sig | sig |
| Duration of epilepsy | Histopathology diagnosis | 3.94E-12 | sig | sig |
| Duration of epilepsy | Seizure outcome | 1.90E-01 | ns | ns |
| ASM pre-op | ASM trialed | 2.20E-16 | sig | sig |
| ASM pre-op | Pre-op MRI side | 2.14E-01 | ns | ns |
| ASM pre-op | Pre-op MRI diagnosis | 2.20E-16 | sig | sig |
| ASM pre-op | Procedure type | 1.68E-15 | sig | sig |
| ASM pre-op | Hemisphere operated on | 3.02E-01 | ns | ns |
| ASM pre-op | Lobe operated on | 2.20E-16 | sig | sig |
| ASM pre-op | Histopathology diagnosis | 2.20E-16 | sig | sig |
| ASM pre-op | Seizure outcome | 7.31E-03 | sig | ns |
| ASM trialed | Pre-op MRI side | 5.17E-01 | ns | ns |
| ASM trialed | Pre-op MRI diagnosis | 2.20E-16 | sig | sig |
| ASM trialed | Procedure type | 4.18E-08 | sig | sig |
| ASM trialed | Hemisphere operated on | 6.16E-01 | ns | ns |
| ASM trialed | Lobe operated on | 1.71E-10 | sig | sig |
| ASM trialed | Histopathology diagnosis | 2.20E-16 | sig | sig |
| ASM trialed | Seizure outcome | 8.41E-08 | sig | sig |
| Pre-op MRI side | Pre-op MRI diagnosis | 1.16E-06 | sig | sig |
| Pre-op MRI side | Procedure type | 1.59E-01 | ns | ns |
| Pre-op MRI side | Hemisphere operated on | 2.20E-16 | sig | sig |
| Pre-op MRI side | Lobe operated on | 3.65E-02 | sig | ns |
| Pre-op MRI side | Histopathology diagnosis | 1.25E-04 | sig | sig |
| Pre-op MRI side | Seizure outcome | 1.88E-03 | sig | ns |
| Pre-op MRI diagnosis | Procedure type | 2.20E-16 | sig | sig |
| Pre-op MRI diagnosis | Hemisphere operated on | 5.88E-02 | ns | ns |
| Pre-op MRI diagnosis | Lobe operated on | 2.20E-16 | sig | sig |
| Pre-op MRI diagnosis | Histopathology diagnosis | 2.20E-16 | sig | sig |
| Pre-op MRI diagnosis | Seizure outcome | 2.10E-04 | sig | sig |
| Procedure type | Hemisphere operated on | 9.60E-02 | ns | ns |
| Procedure type | Lobe operated on | 2.20E-16 | sig | sig |
| Procedure type | Histopathology diagnosis | 2.20E-16 | sig | sig |
| Procedure type | Seizure outcome | 6.98E-03 | sig | ns |
| Hemisphere operated on | Lobe operated on | 2.46E-02 | sig | ns |
| Hemisphere operated on | Histopathology diagnosis | 4.06E-01 | ns | ns |
| Hemisphere operated on | Seizure outcome | 3.07E-01 | ns | ns |
| Lobe operated on | Histopathology diagnosis | 2.20E-16 | sig | sig |
| Lobe operated on | Seizure outcome | 2.41E-04 | sig | sig |
| Histopathology diagnosis | Seizure outcome | 3.76E-05 | sig | sig |
| Abbreviations: ASM = Antiseizure medication; Sig. = Significant; Sig. corr = Significant after correction for multiple comparison using the Holm method. | | | | |

| **Supplementary Table 6. Univariable logistic regression analyses investigating predictors of seizure outcome at one-year post-operative follow-up.** | | | | | |
| --- | --- | --- | --- | --- | --- |
|  |  | **OR** | **5-95% CI** | ***P* value** | **Significance** |
| **Demographics** |  |  |  |  |  |
| Sex | Female | Ref | - | - | - |
|  | Male | 1.00 | 0.73-1.37 | 1.000 | ns |
| Handedness | Right | Ref | - | - | - |
|  | Left | 0.74 | 0.51-1.06 | 0.096 | ns |
|  | No clear hand preference/Ambidextrous | 0.31 | 0.16-0.61 | <0.001 | *** |
| Educational status | Mainstream | Ref | - | - | - |
|  | Mainstream with support | 0.61 | 0.38- 0.98 | 0.042 | * |
|  | Not of school age | 0.51 | 0.32-0.80 | 0.004 | ** |
|  | SEN school | 0.33 | 0.20-0.53 | <0.001 | *** |
|  | Home-schooled/Residential home | 0.13 | 0.03-0.43 | 0.001 | ** |
| **Epilepsy characteristics** |  |  |  |  |  |
| Age of epilepsy onset | - | 1.09 | 1.04-1.15 | <0.001 | *** |
| Age at surgery | - | 1.02 | 0.99-1.05 | 0.305 | ns |
| Duration of epilepsy | - | 0.97 | 0.93-1.01 | 0.092 | ns |
|  |  |  |  |  |  |
| **Genetic findings** | No test/Negative result | Ref | - | - | - |
|  | Positive result | 0.24 | 0.13-0.43 | <0.001 | *** |
| **Antiseizure medication (ASM)** |  |  |  |  |  |
| Number of ASM at pre-operative evaluation | 0-2 | Ref | - | - | - |
|  | >3 | 0.68 | 0.49-0.93 | 0.017 | * |
| Number of ASM trialed (from epilepsy onset to surgery) | 0-3 | Ref | - | - | - |
|  | >4 | 0.48 | 0.33-0.70 | <0.001 | *** |
| **Seizure history** |  |  |  |  |  |
| Family history of epilepsy | No | Ref | - | - | - |
|  | Yes | 1.02 | 0.72-1.46 | 0.909 | ns |
| History of febrile seizures | No | Ref | - | - | - |
|  | Yes | 1.00 | 0.65-1.56 | 0.993 | ns |
| History of infantile spasms | No | Ref | - | - | - |
|  | Yes | 0.57 | 0.39-0.85 | 0.005 | ** |
| History of status epilepticus | No | Ref | - | - | - |
|  | EPC | 1.42 | 0.54-4.42 | 0.506 | ns |
|  | Non-convulsive | 0.71 | 0.20-2.80 | 0.598 | ns |
|  | Convulsive | 0.72 | 0.46-1.13 | 0.147 | ns |
|  | Mixed | 0.66 | 0.21-2.26 | 0.486 | ns |
| History of generalized tonic-clonic seizures | No | Ref | - | - | - |
|  | Yes | 0.71 | 0.51-0.98 | 0.036 | * |
| **Seizures at time of pre-operative evaluation** |  |  |  |  |  |
| Number of seizure types | 0-1 | Ref | - | - | - |
|  | 2-3 | 0.56 | 0.40-0.78 | <0.001 | *** |
|  | 4+ | 0.36 | 0.17-0.77 | 0.008 | ** |
| Spasms | No | Ref | - | - | - |
|  | Yes | 0.56 | 0.38-0.83 | 0.003 | ** |
| Focal to bilateral seizures | No | Ref | - | - | - |
|  | Yes | 0.65 | 0.46-0.92 | 0.015 | * |
| Focal vs. generalized onset | Focal | Ref | - | - | - |
|  | Generalized/Mixed | 0.60 | 0.39-0.93 | 0.021 | * |
| Motor vs. non-motor onset | Non-motor | Ref | - | - | - |
|  | Motor/Mixed | 0.82 | 0.48-1.36 | 0.458 | ns |
| Aware vs. impaired awareness onset | Aware | Ref | - | - | - |
|  | Impaired/Mixed | 0.37 | 0.16-0.76 | 0.011 | * |
| Semiology | Localizing-concordant | Ref | - | - | - |
|  | Lateralizing-concordant | 0.92 | 0.58-1.48 | 0.729 | ns |
|  | Non-concordant | 0.65 | 0.44-9.77 | 0.034 | * |
| EEG interictal | Localizing-concordant | Ref | - | - | - |
|  | Lateralizing-concordant | 1.04 | 0.67-1.63 | 0.859 | ns |
|  | Non-concordant | 0.80 | 0.53-1.22 | 0.299 | ns |
| EEG ictal | Localizing-concordant | Ref | - | - | - |
|  | Lateralizing-concordant | 0.90 | 0.56-1.44 | 0.644 | ns |
|  | Non-concordant | 0.69 | 0.45-1.07 | 0.095 | ns |
| **Pre-operative MRI findings** |  |  |  |  |  |
| MRI bilaterality | Unilateral abnormalities | Ref | - | - | - |
|  | Bilateral abnormalities | 0.35 | 0.23-0.55 | <0.001 | *** |
| MRI diagnosis | Rasmussen encephalitis | Ref | - | - | - |
|  | Unspecified tumor | 1.03 | 0.13-21.69 | 0.977 | ns |
|  | Unspecified low grade lesion | 0.75 | 0.16-4.08 | 0.717 | ns |
|  | Scarring | 0.64 | 0.20-1.74 | 0.412 | ns |
|  | LEAT | 0.52 | 0.17-1.40 | 0.226 | ns |
|  | MTS | 0.40 | 0.12-1.10 | 0.095 | ns |
|  | FCD-II | 0.32 | 0.10-0.83 | 0.029 | * |
|  | Negative | 0.29 | 0.05-1.74 | 0.155 | ns |
|  | Vascular | 0.28 | 0.08-0.86 | 0.033 | * |
|  | FCD-NOS | 0.26 | 0.08-0.77 | 0.021 | * |
|  | Not determined lesion/abnormality | 0.22 | 0.07-0.60 | 0.006 | ** |
|  | MCD-Other | 0.22 | 0.07-0.59 | 0.005 | ** |
|  | NSC | 0.17 | 0.02-1.70 | 0.114 | ns |
|  | N-LEAT | 0.17 | 0.02-1.15 | 0.064 | ns |
|  | Tuberous sclerosis | 0.11 | 0.03-0.34 | <0.001 | *** |
| **Surgery information** |  |  |  |  |  |
| Type of surgery | Hemispherotomy | Ref | - | - | - |
|  | Lobectomy | 0.53 | 0.34-0.84 | 0.007 | ** |
|  | Lesionectomy | 0.51 | 0.33-0.78 | 0.002 | ** |
|  | Disconnection | 0.41 | 0.21-0.82 | 0.010 | * |
| Side operated on | Right | Ref |  |  |  |
|  | Left | 0.81 | 0.59-1.10 | 0.171 | ns |
| Lobe operated on | Hemispherotomy | Ref | - | - | - |
|  | Occipital | 1.59 | 0.42-10.40 | 0.555 | ns |
|  | Temporal | 0.61 | 0.40-0.94 | 0.027 | * |
|  | Parietal | 0.52 | 0.26-1.07 | 0.069 | ns |
|  | Multilobar | 0.42 | 0.24-0.75 | 0.003 | ** |
|  | Frontal | 0.34 | 0.21-0.55 | <0.001 | *** |
| **Histopathology** | Rasmussen encephalitis | Ref | - | - | - |
|  | Scarring | 0.86 | 0.25-2.56 | 0.795 | ns |
|  | FCD-NOS | 0.80 | 0.13-6.41 | 0.808 | ns |
|  | LEAT | 0.77 | 0.24-2.03 | 0.616 | ns |
|  | N-LEAT | 0.76 | 0.15-4.27 | 0.736 | ns |
|  | FCD-II | 0.65 | 0.20-1.74 | 0.415 | ns |
|  | MTS | 0.59 | 0.18-1.68 | 0.348 | ns |
|  | M-MCD | 0.45 | 0.07-3.93 | 0.429 | ns |
|  | Vascular | 0.43 | 0.12-1.41 | 0.178 | ns |
|  | MCD-Other | 0.39 | 0.12-1.09 | 0.089 | ns |
|  | Normal result | 0.25 | 0.06-0.92 | 0.042 | * |
|  | NSC | 0.22 | 0.06-0.63 | 0.008 | ** |
|  | Tuberous sclerosis | 0.19 | 0.06-0.56 | 0.004 | ** |
|  | | | | | |
| Abbreviations: ASM = Antiseizure medication; CI = Confidence interval; FCD-II = Focal cortical dysplasia type II; FCD-NOS = Focal cortical dysplasia not otherwise specified; LEAT = Low-grade epilepsy-associated tumor; MCD-Other = Malformation of cortical development other; M-MCD = Mild malformation of cortical development; MTS = Mesial temporal sclerosis; N-LEAT = Non-low-grade epilepsy-associated tumor; NSC = Non-specific epilepsy-associated changes; SEN = Special educational needs; TS = Tuberous sclerosis. | | | | | |

# Supplementary Figures

**
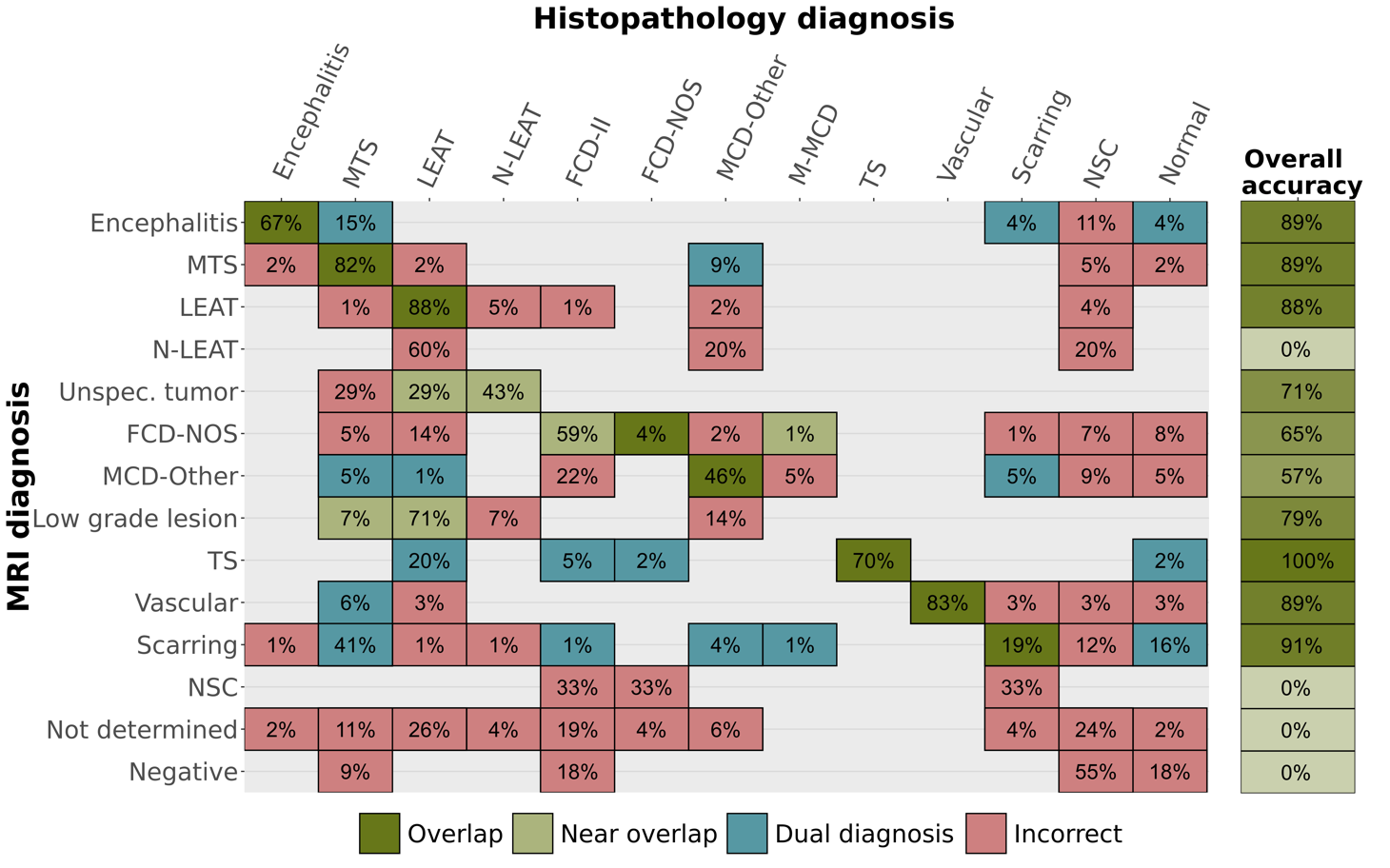
**

**Supplementary Fig. 1.** Overlap between MRI and histopathology diagnoses.

Abbreviations: FCD-II = Focal cortical dysplasia type II; FCD-NOS = Focal cortical dysplasia not otherwise specified; LEAT = Low-grade epilepsy-associated tumor; MCD-Other = Malformation of cortical development other; LGL = Low-grade lesion; M-MCD = Mild malformation of cortical development; MTS = Mesial temporal sclerosis; N-LEAT = Non-low-grade epilepsy-associated tumor; NSC = Non-specific epilepsy-associated changes; TS = Tuberous sclerosis.


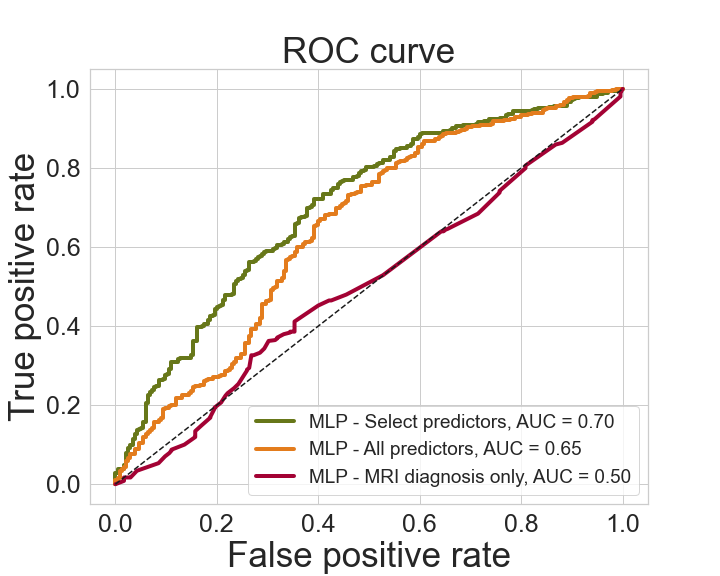


**Supplementary Fig. 2.** Receiver operating characteristic (ROC) curves showing model performance for our MLP model containing 1) only MRI diagnosis (red), 2) all predictors (orange), and 3) predictors identified through data-driven feature selection (green). Data-driven selection involved including only predictors that were significantly predictive of one-year seizure outcome as identified in univariable logistic regression analyses*.*

*
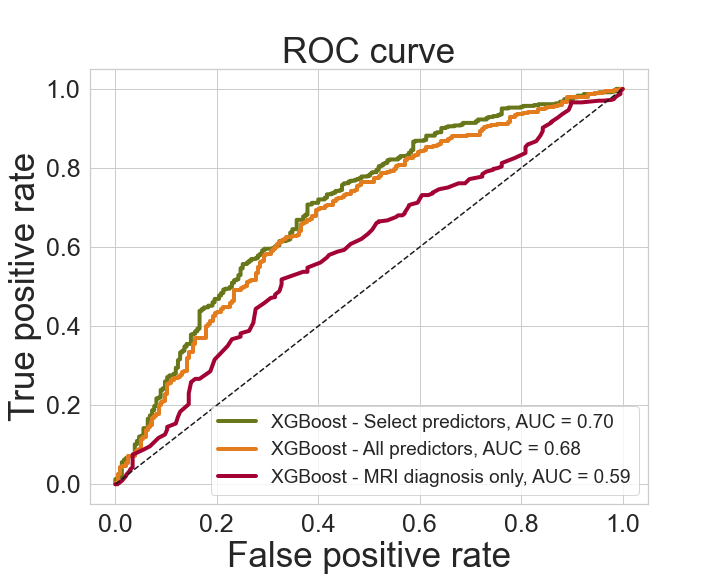
*

**Supplementary Fig. 3.** Receiver operating characteristic (ROC) curves showing model performance for our XGBoost model containing 1) only MRI diagnosis (red), 2) all predictors (orange), and 3) predictors identified through data-driven feature selection (green). Data-driven selection involved including only predictors that were significantly predictive of one-year seizure outcome as identified in univariable logistic regression analyses*.*
